# Zero-dimensional anodes across monovalent and multivalent batteries: a critical review of interfacial dynamics, bottlenecks, and solutions

**DOI:** 10.1039/d6ra08119c

**Published:** 2026-09-30

**Authors:** Harram Najum, Abdul Majid, Muhammad Tayyab Raza, Hameed Zandi, Sheraz Ahmad, Shazia Nawaz

**Affiliations:** a Department of Physics, University of Gujrat Hafiz Hayat Campus Gujrat Pakistan abdulmajid40@uog.edu.pk; b Department of Physics, Gachon University 1342 Seongnam-daero, Sujeong-gu Seongnam-si Gyeonggi-do South Korea ali1214@gachon.ac.kr; c Department of Physics, Quantum Phenomena Research Group, New York University New York City USA; d Dipartimento Di Architettura E Disegno Industriale Via San Lorenzo - Abazia di San, Lorenzo 81031 - Aversa (CE) Italy; e Department of Biological and Chemical Sciences, University of Rasul Mandi Bahauddin Pakistan

## Abstract

The shift towards sustainable energy sources has created an urgent demand for advanced battery systems that can overcome the limitations of conventional anodes. High-capacity candidates such as phosphorus, tin, and silicon-based anodes suffer from poor cyclic stability and severe volume expansion. Higher-dimensional structures such as 1D nanowires, 2D sheets and 3D frameworks may offer high capacity but often suffer from limited scalability, poor structural stability and sluggish kinetics. In contrast, zero-dimensional (0D) nanomaterials provide a unique structural advantage for ion storage. Their exceptionally high surface-to-volume ratio, coupled with quantum confinement effects, shortens diffusion pathways, enhances ion diffusion, improves electrochemical kinetics, and buffers mechanical stress. This review directly compares 0D anode materials across six battery chemistries (lithium-, sodium-, potassium-, magnesium-, aluminum- and zinc-ion systems) with non-0D anode materials in terms of capacity, cycling stability and rate performance, and evaluates them critically. In addition to reporting results, we identify the trade-offs common to 0D architectures across the six systems, namely the balance between capacity gains and first-cycle SEI losses, nanoparticle agglomeration, and synthesis costs that hinder scalability. We emphasize that the evidence does not support a universal advantage of 0D architectures; rather, the benefits depend on the storage mechanism, matrix integration, and the specific failure mode being addressed. We believe that the main remaining challenge is not capacity optimization alone but resolving these trade-offs, which is essential for moving 0D anodes from laboratory demonstrations to commercial batteries.

## Abstract 2

Zero-dimensional anode materials offer high surface area, fast kinetics, structural stability, and high reversibility, which can improve next-generation battery performance and durability.

## Graphical abstract

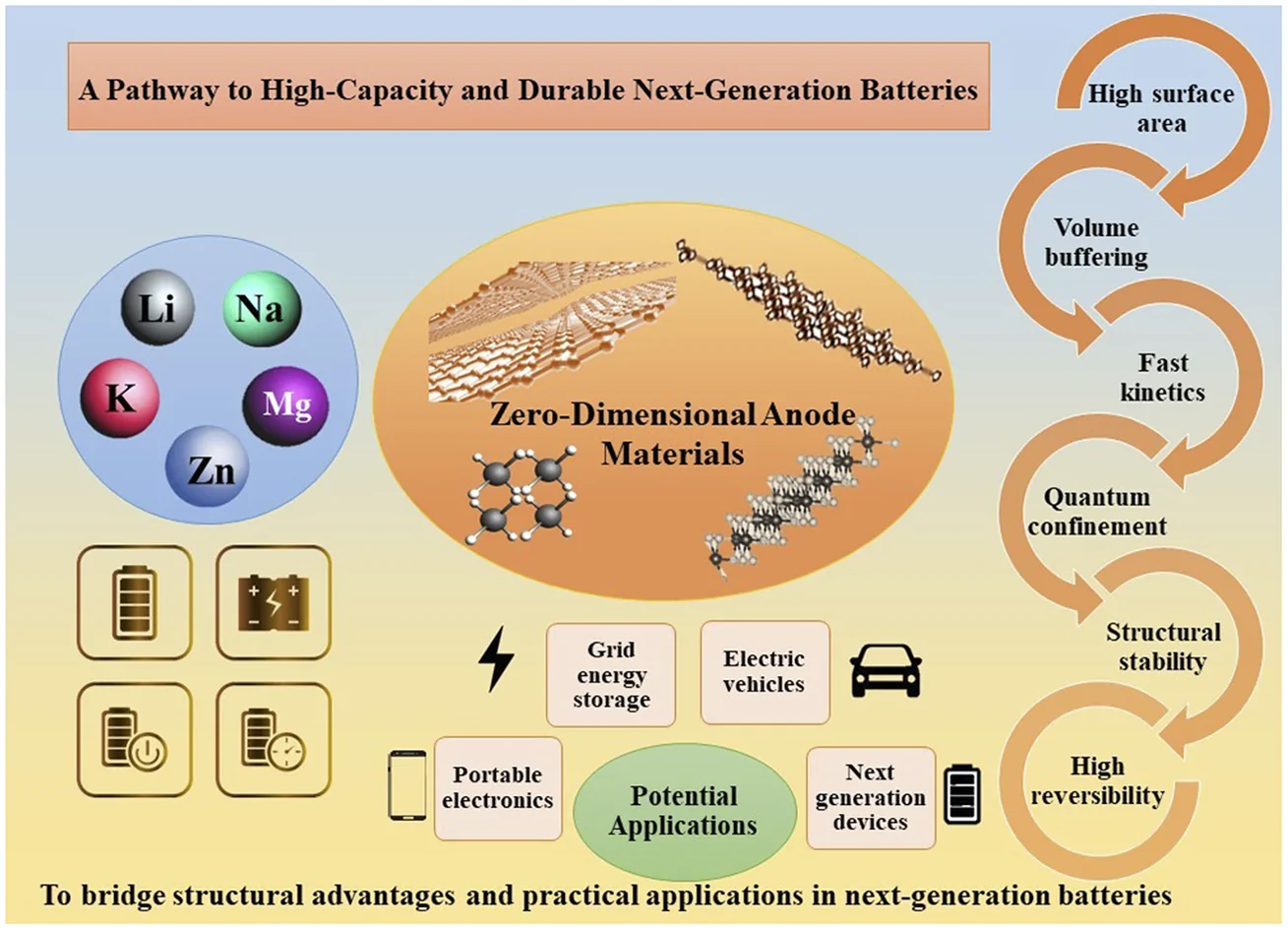

## 1. Introduction

The global energy market is changing dramatically in the 21^st^ century, driven by the urgent need to address environmental concerns and accelerating technological innovation.^1^ The world is steadily shifting toward renewable energy resources such as wind, solar and hydropower as fossil fuels continue to deplete.^1–4^ Growing energy demand has led to an extraordinary rise in the prices of natural resources and petroleum products. Reliance on limited renewable energy resources makes it challenging to produce more efficient and reliable energy storage systems that ensure a consistent energy supply and grid stability.^5–7^ Moreover, the routine use of portable electronic devices, smart technologies and electric vehicles further raises the demand for advanced energy storage solutions that must be highly compatible and environmentally sustainable. Lithium-ion batteries (LIBs), the current benchmark in rechargeable batteries, play a pivotal role in powering portable electronic devices. However, their performance is fundamentally constrained by the characteristics of electrode materials.^8–12^ Rechargeable metal-ion batteries, such as LIBs, NIBs and others, operate on the principle of redox reactions and ion transport. During charging, the cathode releases sodium ions Na^+^ into the electrolyte that then intercalate into the anode. The flow of electrons through the external circuit balances the charge and stores energy. During discharge, the reverse occurs: Na^+^ ions move from the anode into the electrolyte and from there return to the cathode. The connected device draws electrical power from the flow of electrons through the external circuit.^4,13^

The most widely used anode material, graphite, is valued for its affordability, stability, robustness and a theoretical capacity of 372 mA h g^−1^.^14–17^ Despite these advantages, its intercalation mechanism, in which Li ions insert between stacked carbon layers, places fundamental limits on capacity and charge rate. Under fast charging conditions, lithium plating may occur, causing Li to deposit on the surface of the anode rather than intercalate, which can degrade or even damage the battery.^18–21^ To meet the needs of next-generation energy solutions such as large-scale storage and electric vehicles, researchers are actively seeking novel electrode materials and chemistries.^22^ These efforts range from enhancing LIB electrodes to developing systems with non-lithium monovalent and multivalent ions. To overcome the limitations of lithium, such as high cost, safety risks, and limited availability, researchers are looking for alternative materials and technologies.^23–28^ Engineering materials at the nanoscale (1–100 nm) is another powerful route to modify the properties of traditional battery materials.^29^ Reducing the electrode size to the nanoscale enables excellent electrochemical performance, a high surface area, effective ion movement and improved durability.^30–32^ The 0D nanostructured candidates include nanoclusters, quantum dots and nanoparticles, which exhibit electronic and surface properties that differ from those of bulk materials and lower-dimensional (1D/2D) nanostructures because they are confined in all three spatial dimensions.^33–36^ The tunable electronic structure and large surface area enhance electrochemical kinetics but, as this review will demonstrate, these factors also bring trade-offs such as higher side reactivity and SEI growth that are not yet fully understood.^37,38^ This review differs from previous reviews of 0D anode materials in two respects. First, rather than examining one chemistry at a time, it compares the performance gains of 0D confinement across six battery chemistries (lithium-, sodium-, potassium-, magnesium-, aluminum- and zinc-ion batteries) to determine whether the improvements are universal or chemistry-specific. Second, it discusses the reported capacities and cycling performance, along with the recurring costs of the 0D approach: synthesis complexity, nanoparticle agglomeration and first-cycle coulombic losses caused by excess SEI formation. It presents recent advances in the use of 0D nanomaterials as anodes in each of these battery systems in turn. The key structural and electronic properties of 0D nanomaterials (Fig. 2) are discussed first, followed by chemistry-specific sections that assess how these properties can address the challenges of each type of battery. Finally, the review highlights the research gaps that, in our view, hinder the translation of 0D materials from laboratory demonstration to operational devices. The growing research interest in 0D anode materials for batteries is reflected in the publication trend shown in Fig. 1, which covers 2018 to 2026.

**Fig. 1:**
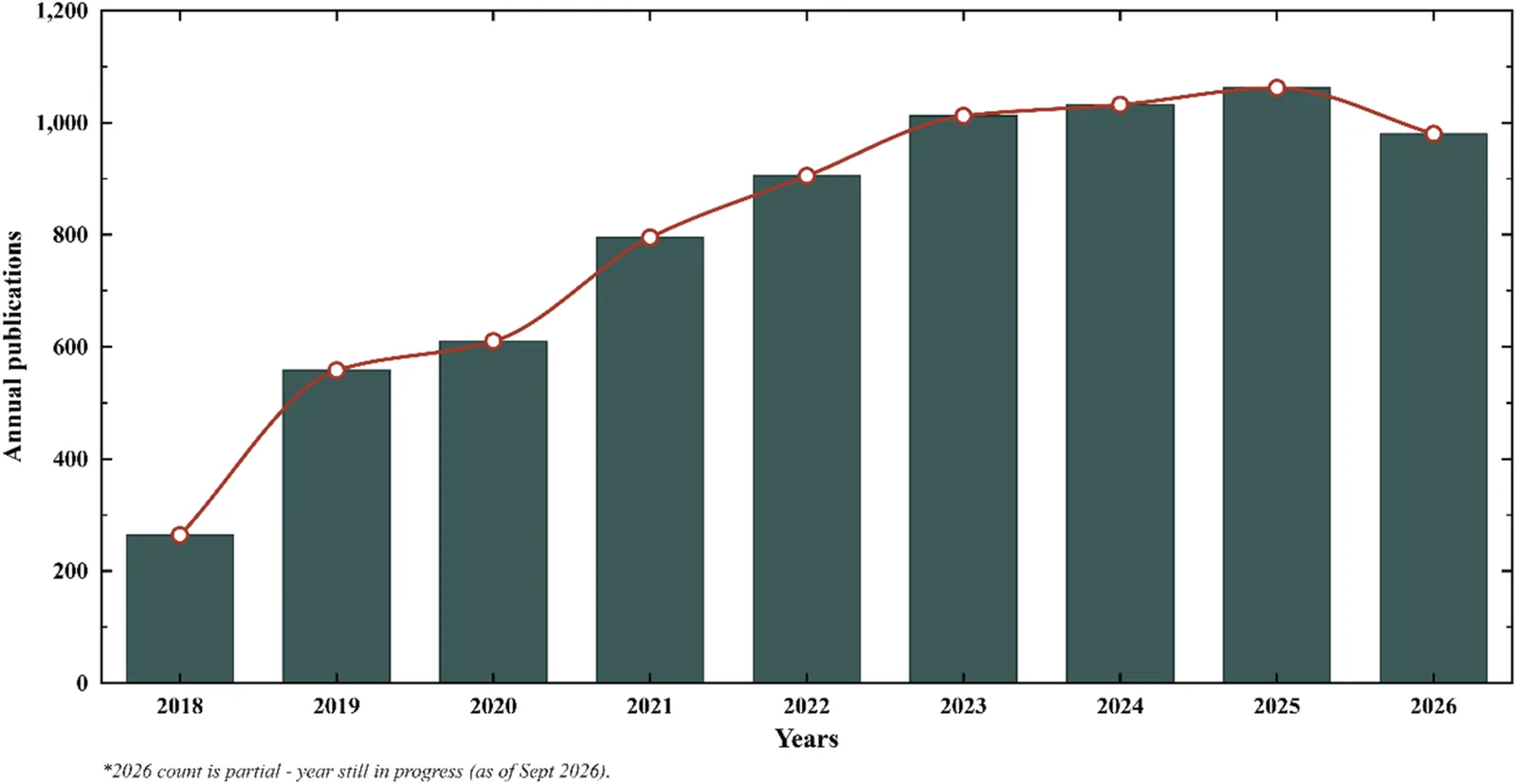
The number of publications annually produced on 0D anode materials for batteries, 2018–2026. The trend confirms that research activity in this field has grown substantially since 2019.

**Fig. 2:**
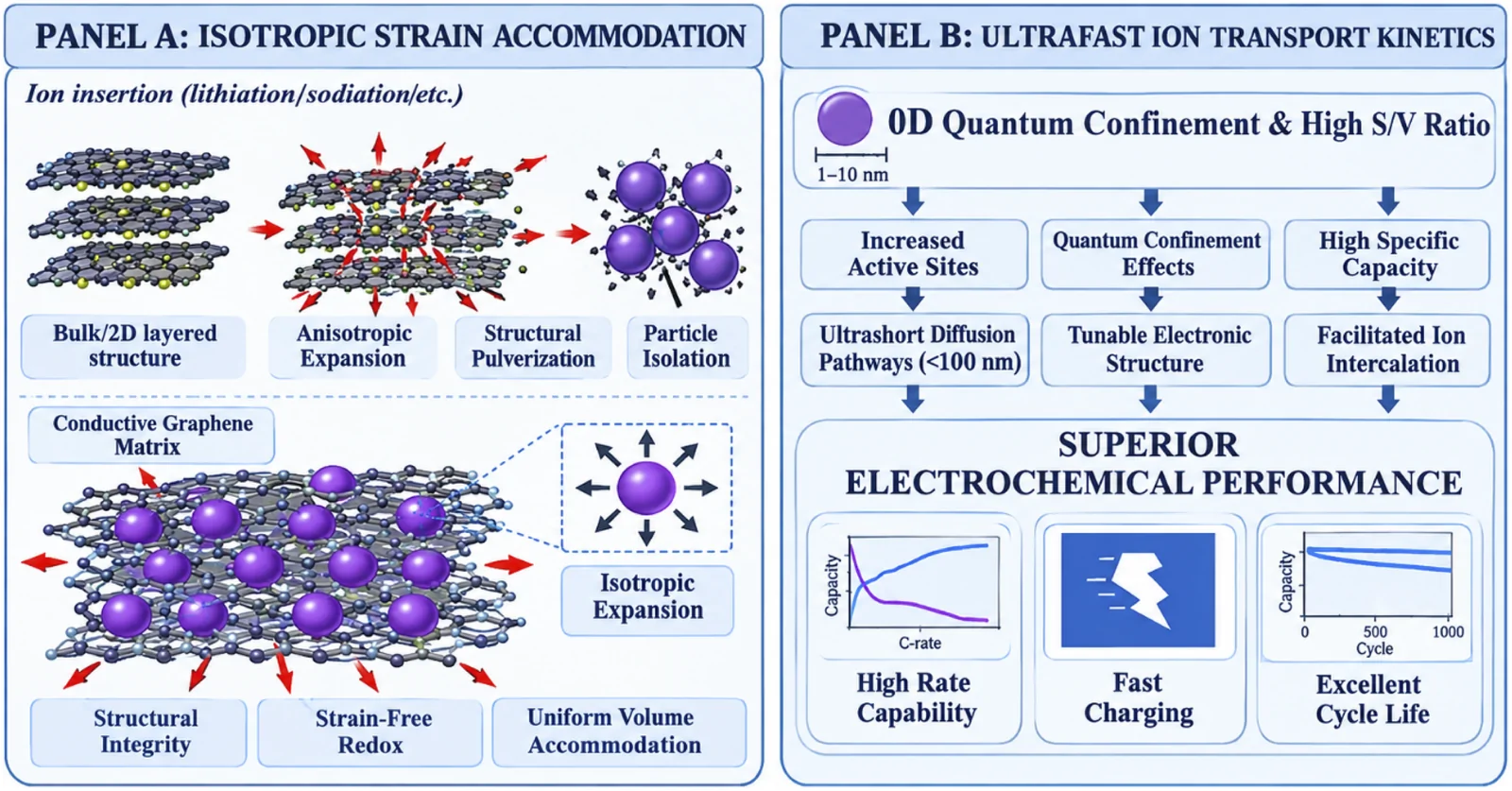
Mechanistic advantages of 0D nanomaterial anodes: strain buffering and enhanced kinetics.

## 2. Zero-dimensional materials: properties and synthesis

### 2.1. Limitations of conventional dimensionalities (3D, 2D, 1D)

The electrochemical reactivity of traditional electrode materials, whether in bulk three-dimensional form, two-dimensional sheets or one-dimensional nanowires, is limited by the inherent anisotropy of strain accumulation during electrochemical cycling and by transport bottlenecks. When ions are inserted into the crystal, the volume change is generally not the same in all directions, and this mismatch creates internal stress gradients that concentrate at grain boundaries, particle surfaces and defect-rich areas. For instance, in silicon-based materials, lithiation may cause a volume expansion of ∼300–400%, and the anisotropic lattice distortion can introduce stress concentrations large enough to initiate cracks and drive their propagation.^35,39–42^

There are three consequences of this mechanical instability. First, fresh reaction surfaces are continually exposed at the fracture, which catalyse electrolyte decomposition and structural degradation.^43,44^ Second, during cycling, the solid–electrolyte interphase (SEI) that grows on the anode surface is repeatedly broken and regenerated, which causes irreversible loss of active ions and a decrease in coulombic efficiency.^18–21^ Third, with progressive cracking, active material particles can become detached from the conductive network (current collector), leading to irreversible capacity loss and electrical isolation.^45–47^

These failure processes tend to be particularly severe in electrodes with large volume changes, *i.e.* those that repeatedly expand and contract. Nanostructuring (*e.g.* 1D nanowires and 2D nanosheets) can moderate these effects, but it does not eliminate them. If one or two dimensions are reduced, the material may be constrained in those directions but can still expand along the remaining direction(s). Furthermore, 1D and 2D structures may introduce directionality in ion transport: ions move easily along the unconfined direction but are blocked in the others.^33–36^ Kinetic bottlenecks therefore remain possible and may limit rate performance and long-term cycling stability. As a result, dimensional reduction has not completely resolved the mechanical and transport limitations of traditional electrode designs.

### 2.2. The 0D advantage: confinement and surface effects

The width, length and height of zero-dimensional nanomaterials are generally below 100 nm, so confinement occurs in all three dimensions. This class comprises quantum dots, nanodots, clusters and nanoparticles made of semiconductors, metals or insulators. The electrochemical properties are influenced not only by particle size reduction but also by nanoscale-specific effects arising from surface chemistry, quantum confinement and interfacial structure. One of the most characteristic features of the 0D nanomaterials is their high surface-to-volume ratio, which provides a large number of active sites for electrochemical reactions. This increased interfacial area ensures good contact between the electrode and the electrolyte and facilitates charge-transfer kinetics, which may enable higher reversible capacities and faster charge–discharge rates, as seen in tin dioxide quantum dots and other 0D electrode materials.^40,48–52^ In addition, the arrangement and mobility of surface atoms are important in determining ion transport and structural stability, and can be tuned through functional groups, surface chemistry and crystallinity to suppress unwanted side reactions and enhance durability.^53–57^

Since 0D structures are completely confined in all spatial directions, the accumulation of anisotropic strain can be reduced compared with extended 1D and 2D structures. The lack of a preferred long axis or extended plane allows, in principle, for a uniform expansion profile when inserting ions, giving a more uniform distribution of strains throughout the particle as opposed to along a single direction. If 0D nanoparticles are incorporated into conductive matrices such as a carbon framework, graphene shells or porous hosts, they can also take advantage of mechanical buffering, in which the surrounding matrix can accommodate the associated volume changes without disrupting electronic connectivity.^53–55^ This strategy has proved helpful in composites such as tin dioxide–graphene systems.^58,59^ Furthermore, the concentration gradients during ion insertion and extraction are diminished owing to the ultrashort diffusion lengths of 0D materials, which can further reduce stress development. Thus, the primary benefit of 0D materials lies not in eliminating side reactions, but in their ability to control kinetics and structural stability simultaneously. The electromechanical distinction between bulk and 0D architectures is shown in Fig. 3. For conventional 2D layered materials, ion intercalation causes anisotropic strain, large interlayer expansion, and lattice fracture, which cause irreversible capacity fading. In comparison, 0D cluster confinement can promote more uniform strain accommodation and reversible expansion/contraction, enabling structural breathing rather than elimination of strain or volume expansion. When appropriately designed and combined with suitable matrices, these features help to maintain structural integrity over long-term cycling.

**Fig. 3:**
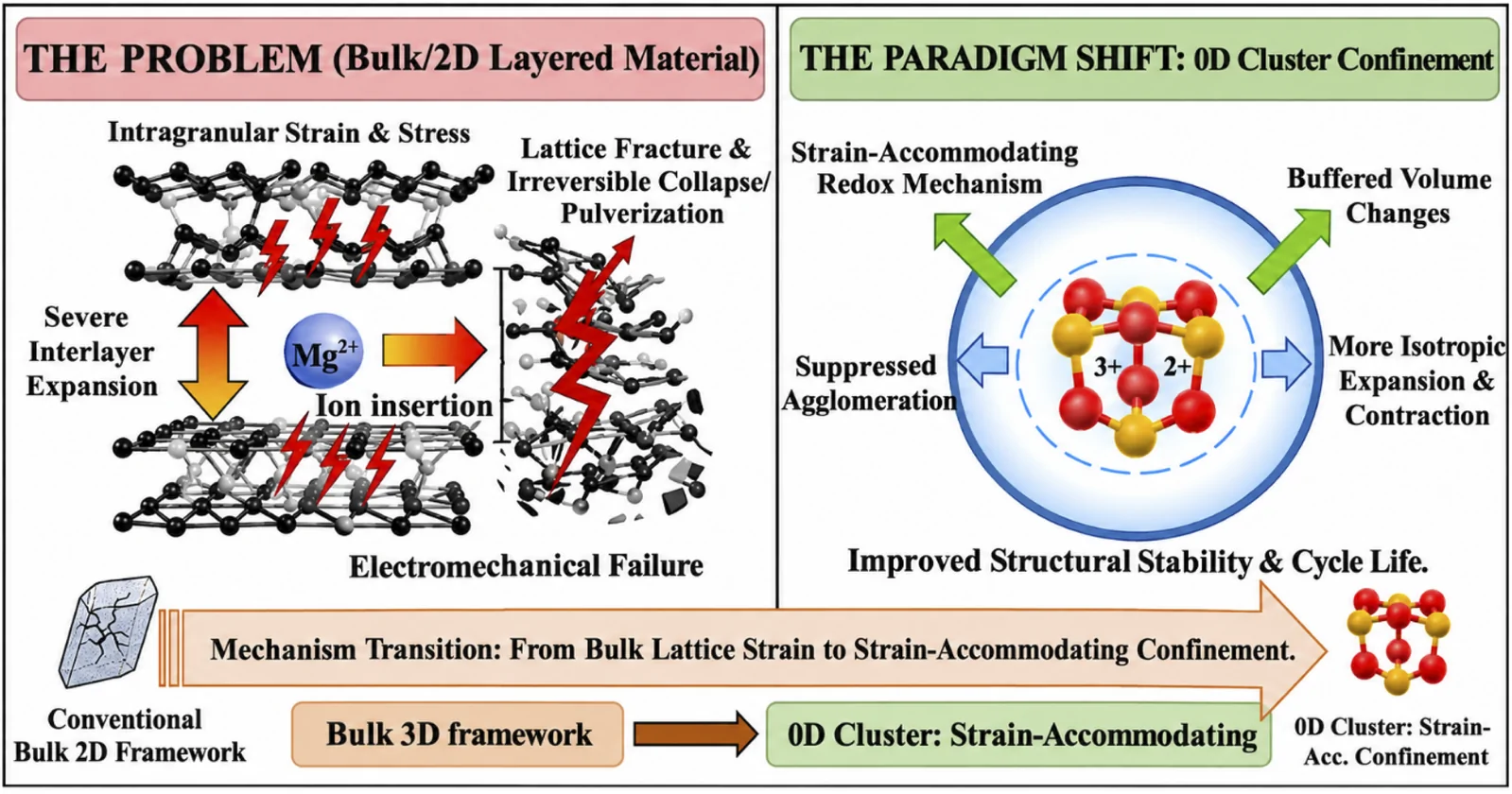
Schematic diagram showing comparison of electromechanical failure in bulk/2D *versus* 0D cluster-confined architectures. In 0D systems, suitable surrounding matrices can more uniformly accommodate and buffer volume changes, enabling reversible structural breathing and improved structural stability.

### 2.3. Synthesis methodologies: precision *vs.* scalability

The electrochemical activity of 0D nanomaterials is closely related to their size, shape, crystallinity, and surface functional groups, which are well controlled by the synthesis method. In general, there are two types of synthesis methods: bottom-up and top-down. A bottom-up approach constructs nanomaterials from atomic and/or molecular building blocks. These methods include physical vapour deposition, hydrothermal synthesis, and chemical and biological methods. Si quantum dots can be fabricated by pyrolysis of a SiH_*x*_O_*y*_ polymer (hydrogen silsesquioxane; H_*x*_O_*y*_ = CH_3_), in which the amount of methanol in the polymer can be used to control the particle size and crystal quality of the Si quantum dots.^58,59^ Likewise, many metal oxide nanoparticles and nanoclusters are synthesized hydrothermally, and the temperature, pressure and precursor concentration can be adjusted to control the size, morphology and phase.

In a top-down approach, the materials are mechanically or physically broken down to the nanoscale. Grinding, mechanical alloying and high-energy ball milling are helpful for scaling up production. For example, phosphorus nanoparticles have been synthesized in carbon matrices using high-energy ball milling for battery applications.^60–62^ The electrochemical properties of 0D materials depend strongly on the synthesis method. The plasma-enhanced chemical vapour deposition process can be used to fabricate crystalline silicon nanoparticles with a narrow size distribution, which are suitable for high-capacity anodes and potentially scalable as well.^63,64^ A significant problem, however, is that many synthesis methods offer limited control over particle size and surface structure at the atomic level. This makes the reported electrochemical performance somewhat susceptible to batch-to-batch variation. Synthesis of 0D nanomaterials therefore needs to be precise, reproducible and scalable to progress towards commercial battery deployment.^65–68^

### 2.4. Computational design and prediction of 0D anode materials

The design and prediction of 0D anode materials depends primarily on first-principles modelling (increasingly aided by machine learning) to screen candidates for their capacity, voltage, conductivity, ion transport, structural stability and interfacial behaviour.^60,69,70^ The main attractions of 0D systems (nanoparticles, nanocages, nanospheres, quantum dots, hollow/core–shell motifs) are their large surface area, high number of ion-storage sites and short transport pathways, but aggregation and interfacial instability remain significant issues.^44^ 0D nanocage and fullerene-type anode candidates have been investigated using a wide range of computational strategies, as summarized in Table 1, which highlights the variety of density functional theory (DFT)-based methods relevant to 0D anode design.

**Table 1:** Representative computational studies directly addressing 0D nanocage and fullerene-type anode candidates

System	Computational strategy	Main predicted outcome	Relevance to 0D anodes	Ref
Pristine and doped Ge_12_C_12_/Si_12_C_12_ nanocages	DFT plus ML regression	Doping with O or Se improved conductivity; pristine Si_12_C_12_ gave 1.49 V and 668.42 mA h g^−1^; Lasso predicted *V*_cell_ with *R*^2^ = 0.99	Direct 0D nanocage anode screening	71
C_24_ fullerene derivatives	DFT hydrogenation study	Hydrogenation preserved Li diffusion and yielded favorable storage behaviour	Direct 0D fullerene design route	71
C_32_ and B_16_N_16_ nanocages	DFT	Predicted cell voltages of 1.03 V and 1.18 V	Direct 0D nanocage comparison	71
C_24_, Si_24_, B_21_N_21_, Al_21_P_21_ nanocages	DFT	Predicted voltages of 0.88, 1.01, 1.24, and 1.43 V	Direct 0D multi-chemistry screening	71
Mg_12_O_12_ and Zn_12_O_12_ nanocages	DFT encapsulation study	Used to evaluate storage capacity effects of F encapsulation	Direct 0D cage functionalization strategy	72

#### 2.4.1 Design principles and computational workflows

Computation is used together with structural and compositional information to relate both to ion uptake, diffusion kinetics, storage efficiency, disorder, pore geometry and interfacial properties, and to guide defect or dopant engineering prior to synthesis for alkali-ion anodes.^60,62^ For 0D candidates, the main limitations in sodium- and potassium-ion systems are low operating potential, a lack of host space for large ions and insufficient mechanical robustness against insertion stresses, so rational nanostructural design is critical.^44,62^ DFT is the central method and is typically followed by adsorption-energy analysis, open-circuit voltage (OCV) and Gibbs free energy calculations, and climbing-image nudged elastic band (CI-NEB) calculations to obtain migration barriers. *Ab initio* molecular dynamics (AIMD) has been used more selectively to evaluate thermal stability, and machine learning is beginning to be used for high-throughput screening.^62,73^

Attractive 0D materials consist of stable hollow or cage-like structures that buffer volume expansion while preserving contact with the electrolyte. However, their electrochemical performance is difficult to realise because of their tendency to self-aggregate, so composite or core–shell designs are preferred.^44^ Overall, first-principles studies of silicon, lithium titanate and lithium-metal anodes show that atomistic calculations are most useful when they illuminate the diffusion kinetics, phase or volume changes and interfacial reaction mechanisms that are difficult to observe experimentally under operating conditions.^62,65–68^

#### 2.4.2 Electrochemical predictions: capacity and voltage

Capacity and voltage are predicted using electrochemical equations. The theoretical capacities reported in 0D first-principles studies span a wide range. DFT predicts a theoretical capacity for pristine Si_12_C_12_ nanocages of 668.42 mA h g^−1^ for LIBs, and for Nb_2_CO_2_ MXene nanoclusters of 960 mA h g^−1^ for SIBs. Some other multivalent-host studies outside strict 0D geometries report even higher values (namely 4700 mA h g^−1^ for Mg in InOCl and 1131–1302 mA h g^−1^ for Al/Mg in lithium titanate oxide^71,74–77^). For 0D molecular cages in Mg batteries, voltage has been discussed more than capacity, which means that the adsorption thermodynamics of such cages is studied more often than the full loading pathway.^78,79^

Open-circuit or cell voltages are high. Pristine Si_12_C_12_ reached 1.49 V in LIB screening, while C_32_ and B_16_N_16_ nanocages were reported at 1.03 and 1.18 V, and C_24_, Si_24_, B_21_N_21_, and Al_21_P_21_ nanocages at 0.88, 1.01, 1.24, and 1.43 V across Li/Na/K-ion systems.^71^ In studies of Mg-ion nanocages, fullerene-like cages have been reported at 2.7–3.7 V. In contrast, a comparison of C_60_ with nanocage studies gives the order cone > tube > sheet > cage for the voltage of an Mg battery cell.^78,79^ The voltages of bare Mg_12_O_12_ and Zn_12_O_12_ were 0.61 and 0.57 V, respectively, but when F^−^ was encapsulated, they increased to 4.05 and 3.55 V, respectively, although Li-doped F^−^@Mg_12_O_12_ at 1.59 V was judged a more acceptable anode candidate.^72^Table 2 summarizes the capacity and voltage predictions from these and related computational studies and allows a straightforward comparison of the electrochemical performance metrics of various 0D systems and chemistries.

**Table 2:** Capacity and voltage predictions across representative nanocage and nanocluster anode candidates. All values in this table are DFT-predicted theoretical values

System	Ion chemistry	Capacity (mA h g^−1^)	OCV	Key note	Ref.
Si_12_C_12_ nanocage	Li	668.42	1.49	Best among tested pristine/doped cages	71
Nb_2_CO_2_ nanocluster	Na	960	—	Oxygen passivation improved stability	74
C_32_/B_16_N_16_ nanocages	Li/Na/K	—	1.03/1.18	Early multi-ion nanocage comparison	71
C_24_, Si_24_, B_21_N_21_, Al_21_P_21_ cages	Li/Na/K	—	0.88–1.43	Multi-chemistry cage screening	71
Mg_12_O_12_/Zn_12_O_12_ cages	Li	—	0.57–4.05	Strong voltage tuning by halide encapsulation	72

#### 2.4.3 Transport and stability: diffusion barriers and AIMD

The most explicit transport workflows extract minimum-energy migration paths with CI-NEB. Ion transport kinetics is a key target of DFT studies; in current studies of candidate anode materials, the adsorption energy is calculated first, followed by the OCV and then the diffusion barriers along candidate paths.^71,75,76^ Calculations of Li storage in doped C_2_N were performed by CI-NEB with five intermediate images, which gave a Li barrier of about 0.36 eV compared with that of pristine C_2_N.^76^ A few examples show how ion charge and host topology alter kinetic barriers: Li (0.20 eV) and K (0.28 eV) both have lower barriers in lithium titanate oxide than Ca (1.51 eV) and Mg (0.43 eV), while Al has a lower barrier (0.44 eV) in InOCl than Li (0.80 eV).^80^

AIMD is rarely used, but when it is, it tends to test thermal stability rather than electrochemical cycling. Different framework materials call for different thermodynamic controls in the simulations used to confirm stability and transport properties. The C_2_N system was explored with the NVT ensemble for 10 ps. For lithium titanate oxide, AIMD was used to demonstrate thermal stability explicitly, and standard molecular dynamics simulations were performed at temperatures from 300 to 900 K to determine the respective diffusion coefficients.^76,81^ The thermal stability of 0D carbon nanorings has also been verified by AIMD simulation up to 1000 K (Fig. 4), which shows that they remain structurally stable at high temperatures.^82^ In some studies, such as those on Na storage, the stability of nanoclusters is inferred from energetic, dynamic and thermal stability criteria instead of long AIMD trajectories. MXene nanoclusters were reported to be energetically, dynamically and thermally stable, particularly when oxygen-passivated. In practice, MD can recover barriers close to NEB values if it is well converged, but it may require high-temperature, long-time sampling, which is why many 0D studies stop at NEB.^77^

**Fig. 4:**
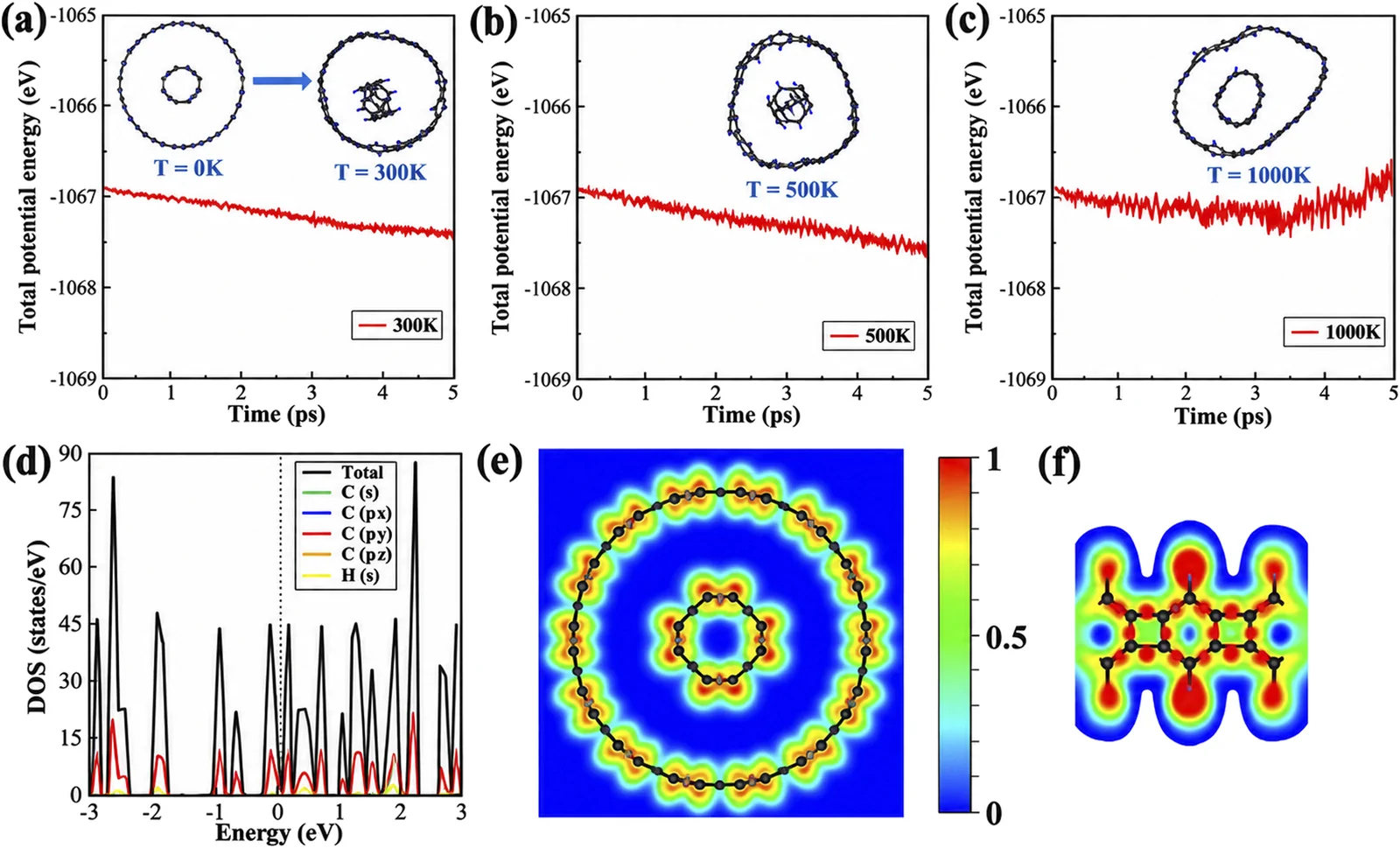
AIMD stability and electronic properties of BPNCRs as 0D anode. (a–c) Total potential energy variation at 300 K, 500 K, and 1000 K. (d) Projected density of states with metallic behaviour. (e and f) Bonding as seen in the electron localization function. This figure has been reproduced from ref. 82 with permission from Royal Society of Chemistry, copyright 2025.

#### 2.4.4 Machine learning and high throughput screening

This area of machine learning is still developing. To predict the cell voltage, the most direct 0D model used DFT-derived descriptors of pristine and O/Se-doped Ge_12_C_12_ and Si_12_C_12_ nanocages and trained LR, Lasso, Ridge, and ElasticNet regressors, with Lasso giving the best *R*^2^ of 0.99 and an RMSE < 0.05.^71^ On a much larger scale, first-principles battery modelling is increasingly combined with automated simulation, data mining and ML, although most available examples of battery screening concern other 0D components rather than 0D anodes.^62,83^ At an even larger scale, a pipeline of ML and HPC screened 32 million candidates and predicted ∼500 000 potentially stable materials, with experimental follow-up in battery-relevant systems, and scaled graph networks found 2.2 million structures below the convex hull, of which 736 had already been realised experimentally.^83,84^ For battery anodes in particular, quantum-assisted ML screened 9835 structures, identified 211 thermodynamically stable ones and correlated stability, diffusion and mechanical properties to rank promising LIB anodes. For migration-barrier screening, high-throughput NEB with fine-tuned CHGNet demonstrates how the computational cost can be lowered without loss of NEB/MD-level insight.^85^

#### 2.4.5 Capabilities and limitations of computational predictions

DFT, AIMD and machine learning are worthwhile tools for screening 0D anode candidates. DFT-calculated adsorption energies, open-circuit voltages and theoretical capacities provide helpful guidelines for material selection before synthesis. AIMD predicts thermal stability and diffusion behaviour over finite time scales, whereas machine learning enables high-throughput screening across the available structural databases. Together, these techniques have identified promising 0D candidates, clarified ion-transport mechanisms that are difficult to measure experimentally and supported defect and dopant engineering before synthesis.

However, there are important limitations associated with these theoretical methods. First, the values obtained with these techniques are usually based on idealized, defect-free 0D units, whereas experimentally synthesized materials are polydisperse, contain surface defects and may be amorphous or partially crystalline. Second, adsorption-energy and OCV calculations assume idealized ion-insertion channels and ignore interfacial charge-transfer resistance and electrolyte decomposition, which dominate first-cycle efficiency. Third, CI-NEB migration barriers describe intrinsic host kinetics rather than the rate capability of a device.^88^ For example, the barriers for Li (0.20 eV) and K (0.28 eV) are lower than those for Ca (1.51 eV) and Mg (0.43 eV), but these values refer to isolated diffusion paths and do not account for particle agglomeration or electrode tortuosity.^82^ Fourth, band gaps calculated with GGA-PBE are usually underestimated, so the predicted voltages can differ by several tenths of a volt from hybrid-functional or experimentally measured values.^89^ These uncertainties are rarely reported as error bars in the primary literature, which contributes to deviations between theory and experiment. Nevertheless, these constraints do not lessen the importance of computational screening; they mark the boundary between theoretical prediction and experimental validation. The DFT-calculated capacities in Table 2 should therefore be read as theoretical upper bounds that motivate synthesis, while the experimental values in Tables 3–6 serve as practical benchmarks.

## 3. Mechanistic classification of 0D anodes

0D anode materials across the six chemistries surveyed here do not share a single storage mechanism.^90^ Treating them as a uniform category may therefore cause confusion rather than clarify their comparative performance. Four mechanistic classes must be distinguished. (i) Intercalation hosts such as TiO_2_, Nb_2_O_5_, graphite and related layered oxides store ions through lattice insertion without any phase change. The failure mechanism of 0D anodes in this class involves anisotropic strain accumulation.^91^ (ii) Alloying hosts such as Si, Sn, Sb, and P store ions by forming reversible alloys, which can cause significant volume changes during cycling. In these systems, 0D confinement mainly provides shorter ion diffusion pathways and helps accommodate the large volume changes associated with alloying. However, it does not completely remove the strain generated during the alloying reaction.^92,93^ (iii) Conversion-type hosts such as Co_3_O_4_, Fe_3_O_4_ and sulfide/selenide QDs undergo phase decomposition on cycling. Here, 0D confinement primarily shortens diffusion length and buffers the larger volume changes intrinsic to conversion reactions, but does not eliminate the irreversible phase change itself. The capacity fading in this class is therefore governed as much by SEI chemistry as by particle geometry.^93^ (iv) Metal-deposition anode systems such as Zn and, to a lesser extent, Mg and Al do not store ions by insertion; instead, the anode itself is the redox-active metal. 0D materials such as CQDs and MXene QDs function as interfacial modifiers of nucleation and plating rather than as storage hosts.^94^ The performance of materials in classes (i), (ii) and (iii) is based on ion storage mechanisms, whereas class (iv) operates through an interfacial mechanism. The collapse of these four classes into a single 0D anode category, as much of the prior literature does, is a primary source of inconsistent benchmarking. The sections and tables below are organized to keep this distinction explicit. Section 4 discusses whether 0D confinement is a universal or a class-specific advantage.

### 3.1. Lithium ion batteries (LIBs)

The concept of ion intercalation into layered structures such as metal compounds and graphite represented a significant advance in the development of LIBs.^8^ Innovation in nanomaterials continues to drive LIB improvement, with dimensionality (0D, 1D, 2D and 3D) emerging as a key design axis for next-generation electrode materials.^45^ To enhance lithium storage capacity and electrochemical activity, 0D nanomaterials have become a research focus, and hybrid/complex structures are being developed to address rate performance, structural stability, and reaction dynamics. 0D nanomaterials attract attention for their large surface area, short ion-diffusion length, and tunable electronic characteristics, which are valuable for both current and future battery systems.^46,86^ Recent developments in LIB chemistry include polymer electrolytes, organic materials, cathode/anode improvements, and other cutting-edge chemistries. Refined synthesis, crystal chemistry, structure–property relationships, redox behaviour and physicochemical characterization are all necessary for these advances. Before discussing individual materials, it is important to note that a “good” 0D LIB anode result should be close to or better than the 372 mA h g^−1^ of graphite, retain most of that capacity after ∼100 cycles and maintain a coulombic efficiency of >∼95% after the first few cycles (if the efficiency in the first few cycles is below this threshold, the SEI is likely unstable or agglomeration is occurring, regardless of the headline capacity). The studies below are therefore judged not only by their initial capacity but also against this benchmark.^86–88^ To increase the longevity, operational effectiveness, and sustainability of nanomaterials for LIBs, researchers are actively investigating both theoretical and practical techniques. Recent 0D anode materials have received considerable attention in this respect because they improve the cycling stability and lithium storage capacity.^89–92^Fig. 5 illustrates the delithiation/lithiation mechanism of SnO_2_–Co_3_O_4_ as a conversion-type mechanism. During discharge, Co_3_O_4_ and SnO_2_ undergo a conversion reaction forming metallic Co and Sn trapped in a LiO_*x*_ matrix, while during charge, Li–Sn de-alloys back to Sn. The combination of both reactions yields high specific capacity, though nanoscale engineering is still needed to suppress volume change and maintain cyclic stability.

**Fig. 5:**
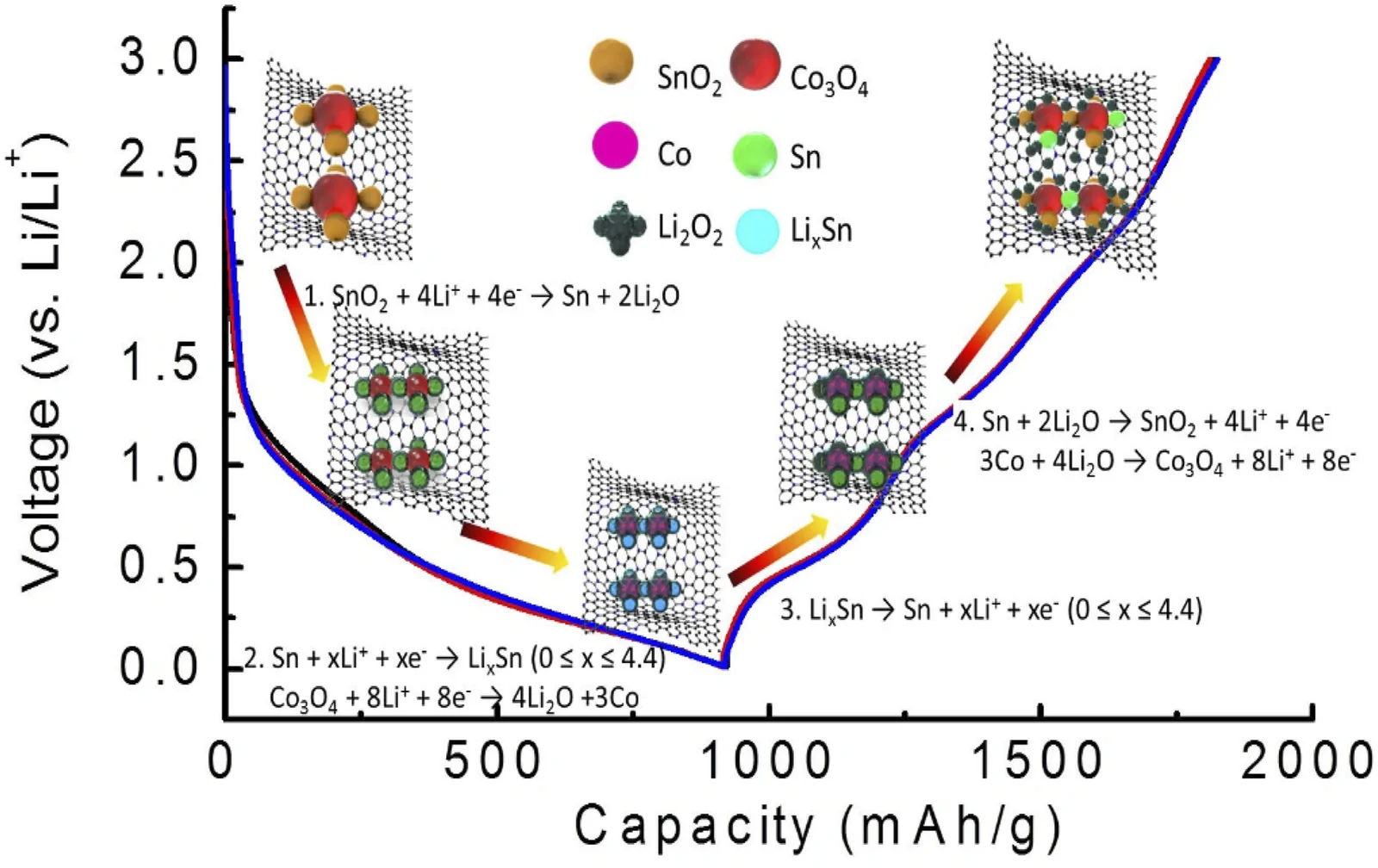
The mechanism of lithiation and delithiation of SnO_2_/RGO/Co_3_O_4_ nanocomposites: the Co_3_O_4_ converts to Co, Sn alloys/dealloy with Li. This figure has been reproduced from ref. 94 with permission from Springer Nature, copyright 2015.

The performance of 0D anodes in LIBs can best be understood by grouping them according to their storage mechanism: alloying, conversion or intercalation. This means examining the effect of 0D confinement on ion transport, electronic conductivity and strain accommodation within each group, rather than simply presenting a single list of capacity values. Controlled hydrothermal processing and annealing yielded uniformly distributed nanocrystals with a mesoporous structure in conversion-type Mo-doped NiCo_2_O_4_, where the conductivity was found to depend on the Mo doping level.^102^ By the above criterion, this result is strong because the capacity retention and CE values exceed the benchmark despite the reduction in capacity from 1225 mA h g^−1^ (at 50 mA g^−1^) to 512 mA h g^−1^ (at 300 mA g^−1^) as the current density increases. The superior electrochemical performance of LIBs has made silicon-based anode materials increasingly attractive.

Silicon-based anodes (alloying-type) remain especially attractive for their high theoretical capacity. Silicon based nanoparticle (SiNP) and nanographite (SiNG) composites as anode materials for LIBs, have been investigated as an alternative to conventional anodes. With an outstanding coulombic efficiency of 100.48%, the SiNP anode provides a reversible capacity of 1568.9 mA h g^−1^. It drops to 1137.6 mA h g^−1^ after 100 cycles, when a stable Li–Si alloy forms. At 8400 mA g^−1^, a stable rate performance of 1191 mA h g^−1^ was maintained owing to the carbon matrix. In contrast, the SiNG anode provided an initial capacity of 1780.7 mA h g^−1^ and maintained 1297.5 mA h g^−1^ with a somewhat elevated resistance after 100 cycles. These results demonstrate how carefully designed 0D silicon-based nanocomposites can improve the stability and electrochemical efficiency of LIB anodes.^93^ Recently investigated 0D anode materials for LIBs are summarized in Table 3. These materials are nanocrystals and nanoparticles; although they are not strictly 0D, their properties are improved by 0D modification.

**Table 3:** True 0D and modified anode materials for LIBs

Anode material	0D structure	Capacity (mA h g^−1^)	Capacity basis	Cycle life	Rate	Key advantage	Ref.
Graphite	—	372	Theoretical	>1000	—	Cheap, stable	14 and 15
Si-QDs	Quantum dots	1957	Initial	100	200 mA g^−1^	Very high initial capacity	100
Carbon coated Si_5_O	Nanoparticles	2558	Initial (89% CE)	—	—	Ultra-high capacity	104
GQD/SiO_*x*_/C	QDs in carbon	595	After 100 cycles	100	0.1/2C	Good retention	97
SnO_2_/graphene	Nanoparticles	634	After 50 cycles (98% CE)	50	—	Buffers volume expansion	95
SnO_2_-QDs/N-doped carbon	QDs in carbon	1255	After 100 cycles (72.9% CE)	100	0.1 A g^−1^	High capacity, N doping	96
Bi_2_O_3_–CQDs	QDs composite	1500	At 0.2C	—	0.2C	Low-cost synthesis	98
SiNP/SiNG	Nanoparticles	1568.9/1780.7	Initial (100% CE)	100	—	Nanographite boosts conductivity	93
Fe_2_O_3_ QDs	Quantum dots	1582.5	Initial	110	1 A g^−1^	Buffers volume changes	102
MoS_2_ QDs	Quantum dots	∼1100	—	—	—	Excellent rate performance	105
Co_3_O_4_ QDs	Quantum dots	1785	Initial	—	—	Suppressed volume expansion	103
SiO_2_ QDs/rGO	QDs cluster	865	Initial (99% CE)	60	—	High initial capacity	99

Among these, SnO_2_ (conversion-type) anode materials are well known for their high theoretical capacity in LIBs. Despite their promise, low coulombic efficiency and poor stability prevent their practical application. SnO_2_/graphene hybrid nanoparticles were prepared by a cost-effective chemical route to address these issues. SnO_2_ nanocomposites perform well as a durable, high-capacity anode material for LIBs. The anode maintained a 634 mA h g^−1^ discharge capacity and 98% coulombic efficiency after 50 cycles. Graphene nanosheets, which serve as a mechanical buffer and reduce volume expansion during lithiation and delithiation, are responsible for this exceptional reversibility.^95^ To further enhance the electrochemical performance and cycling life of LIB anodes, more efficient strategies were adopted. *In situ* polymerization and hydrothermal processing were used to incorporate SnO_2_ QDs into a nitrogen-doped porous carbon matrix. The small size of the QDs improves reaction kinetics and coulombic performance by shortening transport routes and suppressing structural stress. With a coulombic efficiency of 72.9%, SnO_2_ reaches a high capacity of 1255 mA h g^−1^ at 0.1 A g^−1^ after 100 cycles and 753 mA h g^−1^ after 1500 cycles.^96^ To overcome the inherent difficulties of higher-dimensional materials, another 0D anode material has been developed. Graphene QDs and carbon nanoparticles containing SiO_*x*_ were combined to create the GQD/SiO_*x*_/C composite (alloying-type). Internal cavities created by the carbon nanoparticles improved structural stability while also reducing SiO_*x*_ volume variations, as shown in Fig. 9(e) and (f). At 0.1/2C, it delivers its initial capacity of 595 mA h g^−1^ with 92% capacity retention and 81% rate performance. This strategy demonstrates the effectiveness of SiO_*x*_-based anodes for LIBs.^97^

A Bi_2_O_3_–CQD composite (conversion-type) was prepared in a recent study by hydrothermally generating the CQDs from spoiled milk. This 0D composite has outstanding lithium storage capability, achieving a remarkable 1500 mA h g^−1^ discharge capacity at 0.2C. CQDs are essential for increasing electrical conductivity and lowering interfacial resistance.^47,98^ Low-temperature solution-based synthesis was used to incorporate SiO_2_ QDs into rGO films (alloying-type). Lattice fringes of 0.312 nm and 0.277 nm correspond to the (101) and (110) atomic planes, respectively, and the crystallinity was verified by the HR-TEM image in Fig. 7(a). This electrode delivers initial discharge capacities of 865, 417 and 398 mA h g^−1^ and charge capacities of 389, 371 and 363 mA h g^−1^ at a current density of 51 mA g^−1^. After 60 cycles, it retained a capacity of 29 mA h g^−1^ with a coulombic efficiency of 99%. Additionally, the electrode exhibits an outstanding rate capability of 214.4 mA h g^−1^ at 1.02 mA g^−1^, demonstrating the resilience of the material under charge–discharge conditions (Fig. 7(b)–(d)).^99^ Choi *et al.* reported that conjugated compounds help to produce better electrochemical performance (alloying-type). After 100 cycles at 200 mA h g^−1^, they found that the bridge-structured Si QDs preserved 63% of their initial discharge capacity of 1957 mA h g^−1^. During alloying, they help to maintain structural integrity and prevent conductivity loss caused by mechanical stress. Adding specific functional groups and tuning the organic structure could improve the performance of the Si-QD clusters even further.

These findings present a promising path for the development of high-performance anode materials for LIBs.^100^ Owing to its fast rate capability, structural stability and chemical stability, Ca_3_Co_4_O_9_ (intercalation-type), a misfit-layered thermoelectric compound, has gained popularity for use in next-generation batteries. Using molten-salt and co-precipitation procedures, 0D polyhedra of Ca_3_Co_4_O_9_ were prepared with diverse morphologies. To investigate the effects of dimensionality on the material, a thorough electrochemical analysis was carried out. Even after 100 cycles, they demonstrated an exceptional 316 mA h g^−1^ capacity with a coulombic efficiency of over 95%. A large number of reactive sites reduces resistance and volume stress and makes it easier to insert and extract Li ions. The practical application of Ca_3_Co_4_O_9_ in high-efficiency battery technologies can be expanded by these nanoscale engineering strategies.^101^ By carefully controlling the DMF-to-water ratio, nano-Fe_2_O_3_ particles were deposited electrochemically on a nickel substrate (conversion-type). The electrode material for LIBs was then prepared by a second electrodeposition step to deposit graphene QDs as the base for Fe_2_O_3_ composites. An electrochemical study of the anode in LIBs confirmed its exceptional cycle stability and rate capability. They achieved a measured capacity of 1320 mA h g^−1^ (110 cycles) at 1 A g^−1^, as well as a high capacity of 1582 mA h g^−1^. Even at 5 A g^−1^, the anode maintains 1091 mA h g^−1^. These findings demonstrate Fe_2_O_3_/GQDs composites' potential as high-performance LIB anode materials.^102^ Although they are promising anodes, transition metal oxides suffer from low cycle stability and significant volume expansion. The Se-QDs of Co_3_O_4_ (conversion-type) were prepared using a simple chemical method in which Co_3_O_4_ is inside a graphene shell. The graphene shell suppresses volume expansion. Compared with pristine QDs, they exhibited a reversible capacity of 820 mA h g^−1^ over 100 cycles at 1000 mA g^−1^. This encapsulated approach is effective, and the increased stability and cycle life of Co_3_O_4_ QDs make them a good anode material for LIBs.^103^

The limitations of traditional non-0D anode materials such as bulk silicon and graphite are well documented. Bulk silicon suffers a volume expansion of about 300%, which results in capacity fading and mechanical degradation during Li intercalation.^14,15^ 1D and 2D anode materials such as silicon face the major issue of dendrite formation, which may be resolved by engineering approaches. However, these engineering techniques are complex and difficult, and success is still not guaranteed. Reshaping the material alone does not resolve the instability and volume expansion issues.^40,49–52,106^ Moreover, tin oxides, in their bulk form, face mechanical stress, low capacity retention and low rate performance. In comparison, 0D silicon quantum dots (Si-QDs) limit the volume changes relative to bulk silicon, with an initial capacity of 1957 mA h g^−1^.^100^ The energy density of 3D/2D graphite anodes is limited by their low theoretical capacity of 372 mA h g^−1^, whereas 0D nanomaterials have substantially improved electrochemical performance. Si-QDs, SiNP/SiNG, SnO_2_-QDs/N-doped carbon, GQD/SiO_*x*_/C, Bi_2_O_3_–CQDs, Fe_2_O_3_/GQDs, and Co_3_O_4_ QDs achieve better rate capability, higher storage capacity, less volume expansion and long cyclic stability, as reported in this section.^93,95–97,102,105^ For example, SnO_2_-QDs/N-doped carbon achieves a high capacity of 1255 mA h g^−1^ with long cyclic stability, while bulk SnO_2_ cannot maintain it.^96^ These advantages are also listed in Tables 3 and 4.

**Table 4:** The benchmarking of 0D materials against non-0D counterparts for LIBs and PIBs

Non-0D anodes	Limitations	True and modified 0D anodes	Advantages
Bulk Si (LIBs)^125^	Fast capacity fading, severe volume changes (∼300%)	Si-QDs/SiNP–SiNG^93^	High reversible capacity (1568.9 to 1780.7 mA h g^−1^), nanographite boosts conductivity and stability
Co_3_O_4_ (LIBs)^126^	Theoretical capacity (∼800–1000 mA h g^−1^), structural instability, volume expansion	Co_3_O_4_ QDs in graphene shell^103^	1785 mA h g^−1^ (initial) or 820 mA h g^−1^ (after 100 cycles at 1000 mA g^−1^)
Fe_2_O_3_ (LIBs)^126^	Volume expansion, low cycle stability	Fe_2_O_3_/GQDs^102^	Improve reversible capacity of 1582.5 mA h g^−1^, buffering volume changes, and high rate stability
SnO_2_ (LIBs)^127^	Uneven cycling, poor coulombic efficiency	SnO_2_-QDs/N-doped carbon^96^	High capacity, dulls volume expansion
Graphite (LIBs)^128^	Low theoretical capacity (∼372 mA h g^−1^)	GQD/SiO_*x*_/C^97^	Improves theoretical capacity (595 mA h g^−1^), good retention
3D Sb (PIBs)	Volume expansion and fast capacity decay due to large K^+^ addition strain, poor cycling stability	Sb@CQD nano-engineered anode^129^	High reversible capacity (∼480 mA h g^−1^ at 0.5 A g^−1^) with ∼95% retention over 350 cycles, improved kinetics, low volume change
3D Bi (PIBs)	Slow kinetics and structural instability with K^+^, poor rate performance	Bi@C (Bi nanoparticles in carbon skeleton)^130^	Reversible capacity ∼225.6 mA h g^−1^ after 200 cycles and excellent high-rate performance, suppress volume changes
Phosphide (SnP_3_) (PIBs)^131^	Significant volume changes due to bulk form, low rate performance and stability	SnP_3_/C nanosized composites^130^	Improve capacity (∼410 mA h g^−1^) over 700 cycles and improved stability during cycling
Sb/CNS composite (PIBs)^132^	Moderate capacity (∼288 mA h g^−1^), low-rate performance due to larger particle domains and diffusion paths	Sb–CNF nanocomposites^133^	255 mA h g^−1^ over 2000+ cycles and improve transport
FeS–C 2D/3D (PIBs)^134^	Larger in size and slower K diffusion	FeS nanodots^135^	∼232 mA h g^−1^ over 3000 cycles due to nanodot confinement and fast surface kinetics
MoSe_2_ with carbon coating (PIBs)^136^	Slow and restacking diffusion	MoSe/C (PMC)^136^	322 mA h g^−1^ over 100 cycles, improved rate performance
SnS_2_ nanosheets (2D) (PIBs)	Fully sluggish, need further refinement	SnS QDs^137^	621 mA h g^−1^, high reversible capacity

Across the LIB materials surveyed, the structure–property relationship and performance mechanism have been found to be consistent. The capacity scales with alloying content (Si > Sn > oxide-conversion hosts), but retention scales inversely with volume change per cycle rather than with capacity itself. Si-QDs deliver the highest initial capacity of 1957 mA h g^−1^ but the steepest relative fade, to ∼63% by cycle 100. On the other hand, the matrix-confined conversion hosts (Co_3_O_4_@graphene and SnO_2_@N-doped-C) sacrifice roughly 30–45% of peak capacity in exchange for substantially better long-term coulombic stability. This indicates that 0D confinement alone is not the performance-determining variable. Instead, the conductive-matrix architecture surrounding the 0D unit appears to be the determining factor, consistent with the mechanistic distinction drawn in Section 3. Despite several advantages, LIBs also face specific limitations when 0D anode materials are used, as reported in the literature. Mitigating volume expansion and improving cyclic stability leads to reduced storage capacity in some cases, such as Si. The effective surface area and lithium availability are reduced by nanoparticle agglomeration.^45–47^ Excess SEI formation slightly reduces the first-cycle efficiency. Sometimes, the synthesis of nanomaterials becomes difficult and costly, which limits their commercial application.^107^

### 3.2. Sodium ion batteries (SIBs)

Among various post-lithium energy storage technologies, sodium-ion batteries (SIBs) have gained significant attention for large-scale energy storage because of their abundance and low cost. Sodium-based systems are more environmentally friendly and are also consistent with modern energy requirements. Despite these advantages, conventional SIB anodes face major limitations, such as a large ionic radius and sluggish kinetics (Fig. 6).^13,108–111^ Recent studies reveal that 0D materials such as molecular cages, QDs and nanoparticle hybrids offer enhanced storage capacity and rate capability and limit volume expansion. Like LIBs, SnO_2_ anode material performs well for SIBs. In one study, SnO_2_ QDs (conversion-type) were integrated into a nitrogen-doped porous carbon matrix through hydrothermal processing along with *in situ* polymerization. The reduced size of QDs suppresses structural stress and shortens transport pathways, enhancing reaction kinetics and coulombic performance. SnO_2_ delivers a sustained capacity of 212 mA h g^−1^ at 1 A g^−1^ over 3000 cycles, confirming the crucial impact of uniform QD size on electrochemical performance.^96,112,113^ 0D materials, including molecular cages, QDs, and nanoparticle hybrids, offer enhanced storage capacity, rate capability, and limited volume expansion. SnO_2_ QDs (conversion-type) are embedded in nitrogen- and sulfur co-doped carbon fibres *via* calcination, freeze-drying and doping techniques. These composites deliver a high-rate capacity of 141 mA h g^−1^ at 20 A g^−1^ for SIBs. Notably, the material retains its capacity over 10 000 cycles at 5 A g^−1^, showing outstanding longevity.^113^ S doping enhances conductivity and lowers the ion binding energy, while the carbon network suppresses volume expansion. The system maintains stable performance for more than 10 000 cycles, compared with only about 3000 cycles for the undoped material. This suggests that sulfur doping provides additional structural stability beyond the effect of 0D confinement alone, possibly by reinforcing the carbon–SnO_2_ interface and helping it withstand the greater strain caused by Na^+^ ions.

**Fig. 6:**
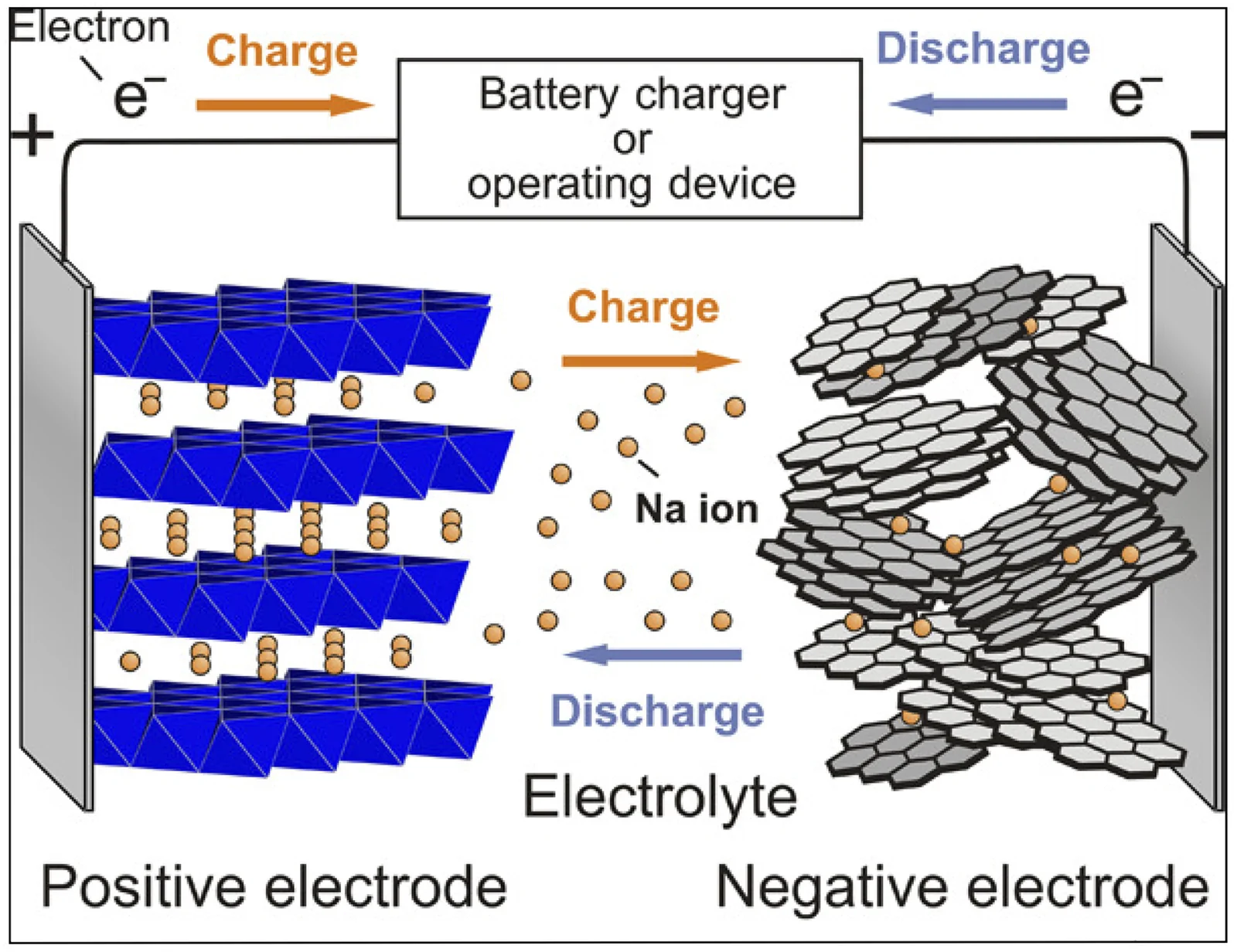
Charging and discharging mechanism of sodium ion batteries. This figure has been reproduced from ref. 13 with permission from American Chemical Society, copyright 2014.

Transition metal oxides (conversion-type) have attracted considerable attention as anode materials for SIBs owing to their high theoretical stability, low cost and improved safety.^114^ A novel anode material, a 3D-0D hybrid structure of graphene and Fe_3_O_4_ QDs, was fabricated by a simple hydrothermal method. This well-designed hybrid structure helps to increase the reaction area, boost electrochemical performance, suppress volume changes and support fast electron–ion transport. As a result, it demonstrates an exceptional storage capacity of 525 mA h g^−1^ at 30 mA g^−1^, excellent rate performance of 56 mA h g^−1^ at 10 mA g^−1^. It also maintains long-term stability of 312 mA h g^−1^ over 200 cycles.^115^ The alloying-type antimony has been analyzed as an N/Sb porous carbon composite (Sb/NPC), which maintains 97% capacity retention at 100 mA g^−1^ after 100 cycles and 357% capacity retention at 1600 mA g^−1^ after 100 cycles, showing carbon confinement benefits.^126^ The comparison between the conversion-type Fe_3_O_4_/graphene hybrid and the alloying-type Sb/NPC composite shows that carbon confinement benefits both systems. However, the alloying-based material delivers a higher overall capacity, whereas the conversion-based system maintains its performance more effectively during cycling. This difference is consistent with the relatively smaller volume changes associated with conversion reactions, as discussed in Section 3.1.

For the first time, researchers presented a novel synthesis route for the fabrication of TiO_2_ nanospheres (intercalation-type) encapsulated in conductive Ti_3_C_2_T_*x*_ MXene layers, which show great promise as an anode for SIBs. The conductive layer preserves the structural integrity and enhances the overall performance of the electrode against electrochemical degradation. The resulting electrode exhibits a moderate capacity of 116 mA h g^−1^ (at 960 mA g^−1^ current density), maintaining performance for up to 5000 cycles. A NaCrO_2_ cathode, when paired with the anode, delivers a full-cell capacity of 103 mA h g^−1^ (960 mA g^−1^) and retains 73.5% of it after 1000 cycles, highlighting excellent cyclic performance.^114^ The concept of integrating 0D nanoparticles with highly conductive materials offers an effective strategy for designing TiO_2_-based anodes. Co-doped amine-modified Ti-MOFs were reported in the literature; they enable the dual doping of N and Co, and ultrafine TiO_2_ nanoparticles were encapsulated in a graphene network. This engineered anode material possessed a reversible capacity of 174 mA h g^−1^ over 5000 cycles and retained 121 mA h g^−1^ up to 3000 cycles. This anode material demonstrated long-term cycling stability and outstanding rate stability compared with many previously reported TiO_2_-based materials.^116^ Vanadium nitride (VN) QDs (conversion-type) were successfully incorporated onto carbon nanofibers (CNFs) to synthesize a novel composite. VN/CNFs were prepared by electrospinning vanadium acetylacetonate as a precursor, followed by nitriding during heat treatment, as shown in Fig. 7(e). The electrode combines the high electrochemical performance of VN QDs with the mechanical robustness of CNFs. VN QDs offer high theoretical capacities but suffer from severe volume expansion. CNFs provide a strong and durable matrix that resists deformation, while the nanoscale QD size promotes faster electrochemical processes. As an SIB anode, the VN/CNF composite delivers stable capacities of 230 mA h g^−1^ after 500 cycles at 0.5 A g^−1^ and 154.7 mA h g^−1^ at 2 A g^−1^ over 1000 cycles (Fig. 7(f)). These findings highlight the potential of VN/CNF composites as a competitive anode for long-life SIBs.^117^ The intercalation-based TiO_2_ systems generally deliver a lower capacity than the conversion-based VN/CNF system. However, TiO_2_ anodes show better capacity retention over 5000 cycles. This improved cycling stability can be attributed to the smaller structural changes associated with intercalation reactions compared with conversion reactions, as discussed in Section 3.

**Fig. 7:**
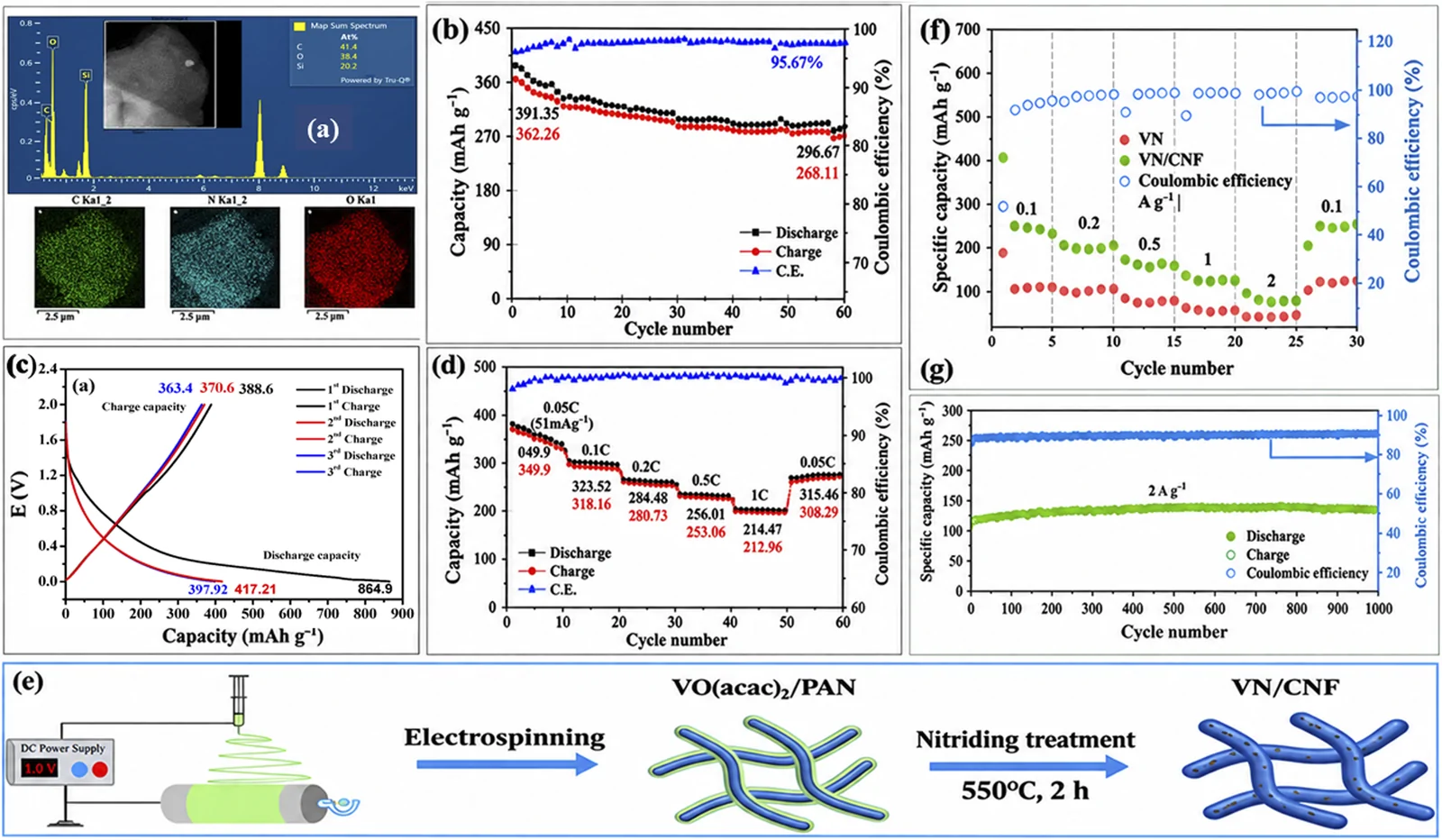
Electrochemical performance of SiO_*x*_/rGO composites (a) HR-TEM image, (b) initial three charge/discharge cycles, (c) cyclic stability, (d) rate capability for LIBs, (e) charge/discharge cycles, (c) cyclic stability, and (d) rate capability for LIBs. VN/CNF composite (e) fabrication of VN QDS on CNFs, (f) rate capability of VN/CNF for SIB, (g) long-term cycling stability at 2 A g^−1^. This figure has been adapted/reproduced from ref. 99 and 117 with permission from MDPI, copyright 2024–2025.

Owing to its ultra-high theoretical capacity of 2595 mA h g^−1^, red phosphorus (alloying-type) has attracted growing attention as an anode for SIBs. However, its practical application is limited by drastic volume changes and low conductivity during cycling, which degrade its electrochemical stability. To overcome such challenges, researchers proposed a novel 0D nanostructure in which nanoscale red P is confined in a nitrogen-doped carbon matrix, forming the PN-MPC 0D composite. This architecture facilitates ion diffusion, boosts conductivity and mitigates volume expansion. This structural design yields a reversible capacity of 600 mA h g^−1^ and maintains 450 mA h g^−1^ over 1000 cycles with minimal degradation of 0.02%, as shown in Fig. 9(c) and (d).^118^ In addition to carbon-based and metal oxide nanoparticles, inorganic molecular cages belong to a unique class of materials. Their discrete, nano-sized, cage-like structures strongly support ion-hosting mechanisms and excellent structural stability and suppress volume expansion. A vanadium-based polyoxometalate (intercalation-type) (Na_6_PV_3_W_9_O_40_) supported on a Co-based metal–organic framework was reported as an anode material for SIBs. A capacity of 413 mA h g^−1^ with 84% capacity retention up to 1000 cycles was achieved, demonstrating its promise for advanced battery systems.^119^ The system comprising red phosphorus shows that the use of a suitable matrix can help achieve its high theoretical capacity. However, the large first-cycle capacity loss and the need for a carefully designed carbon framework suggest that the improved performance originates from the interaction between red phosphorus and the surrounding matrix, rather than from the 0D structure alone.

0D anode materials offer many advantages relative to non-0D anodes across multiple battery systems. For SIBs, clear advantages over traditional non-0D anodes are reported in the literature. An acceptable reversible capacity of 304 mA h g^−1^ is delivered by conventional hard carbon, but it also exhibits a moderate initial coulombic efficiency and low rate performance. This is due to distortion of the carbon structure and slow ion diffusion, which directly affect its electrical conductivity.^112,120^ In contrast, 0D quantum dots and carbon quantum dots improve the performance metrics; for example, nitrogen–sulfur@GCDs deliver a reversible capacity of 384.8 mA h g^−1^ and 155 mA h g^−1^ at 5C. Over 4000 cycles, they still retain 71% of their capacity, which clearly surpasses the rate and stability behaviour of bulk hard carbon.^121^

For NIBs, a novel anode material P_4_Se_3_ has been investigated with a reversible capacity of 654 mA h g^−1^ after 700 cycles at 200 mA g^−1^.^54^ More recently, the cage-like molecular structure of P_4_Se_3_ has been studied using first-principles DFT calculations to investigate its electronic, structural, and ion-storage properties.^133^ The findings indicate a theoretical capacity of 1484.84 mA h g^−1^, suggesting that the molecular P_4_Se_3_ structure has a promising theoretical storage capacity. These results highlight the distinct electrochemical and theoretical characteristics associated with different P_4_Se_3_ configurations. However, direct experimental validation on the material under identical testing conditions is still needed to determine the specific contribution of 0D topology to electrochemical performance. Such studies further illustrate the potential of molecular and nanoscale architectures for SIB anodes, although their experimentally demonstrated performance and structural stability remain to be demonstrated.^133^ Bulk SnO_2_ delivers a theoretical capacity of about 300 mA h g^−1^, and improvements have been found for S-doped SnO_2_ QDs, SnO_2_ QDs embedded in nitrogen-doped carbon, Sn_2_S_3_ QDs in carbon networks and N/S co-doped GCQDs. S-doped SnO_2_ QDs exhibit a capacity of 141.0 mA h g^−1^ at 20 A g^−1^ and maintain 237.0 mA h g^−1^ at 5 A g^−1^ after 10 000 cycles.^123^ Over 3000 cycles, the QDs of SnO_2_ embedded in nitrogen-doped carbon deliver a capacity of 212 mA h g^−1^ at 1 A g^−1^.^106^ Moreover, Sn_2_S_3_ QDs in carbon networks (conversion-type) at 10 A g^−1^ retain a capacity of 300 mA h g^−1^, and at 20 A g^−1^ they maintain 250 mA h g^−1^.^123^ Many more anode materials have been reported, but only a few are highlighted here to illustrate the benefits of 0D nanomaterials.

The benchmarking of 0D against non-0D nanomaterials not only addresses the issues of bulk anodes but also highlights the advantages of 0D anode materials, which achieve high reversible capacity, good rate performance, excellent cyclic stability and low volume expansion. Despite their potential advantages, 0D anode materials for NIBs face several challenges. For example, agglomeration can lower the initial kinetic efficiency of SnO_2_ QDs by reducing the active surface area and Na^+^ accessibility. High surface areas in Fe_3_O_4_ and Sb/NPC QDs can accelerate SEI formation and electrolyte breakdown, which could reduce initial capacity retention.^113^ The ionic radius of Na is large, which slows ion diffusion in the host structure and thereby limits the rate performance of some materials. Moreover, the insertion of Na ions causes volume changes two to three times greater than Li insertion, which can crack even nano-sized particles.^122^ Sample-to-sample variation makes synthesis challenging and affects the electrochemical performance.^107^

For SIBs, the structure–property relationship and mechanism–performance pattern differs from that of LIBs in one respect. The best performance of 0D SIB anodes is obtained when a conversion or alloying host is combined with a carbon-fibre or MOF-based matrix, not from the 0D host alone (*e.g.*, VN/CNFs, S-doped SnO_2_ QDs in N, S-carbon fiber, Fe_3_O_4_ QDs/graphene). The matrix confinement effect also does most of the stabilizing work here compared with LIBs, which is consistent with the greater strain per insertion event that the Na^+^ ion imposes (Section 3).

### 3.3. Potassium ion batteries (PIBs)

Recent advances in 0D nanomaterials have been explored extensively to improve the electrochemical performance of potassium-ion batteries.^26^ However, few studies have examined 0D anode materials for such batteries. Carbon-based materials are identified as potential anode materials for potassium-ion battery systems. Ultrafine N/P co-doped carbon nanoparticles with widened interlayer spacing were engineered within a DFT-guided framework. They exhibit a storage capacity of 270 mA h g^−1^ at 0.2 A g^−1^, while at 1.0 A g^−1^, 190 mA h g^−1^ was achieved after 4000 cycles. Owing to its low volume expansion, DFT-based design strategy and high storage capacity, the NP–CNP anode material paves a new way for designing stable, high-performance carbon-based nanomaterials for PIBs.^123,124^

Hollow nanostructures offer better mechanical stability than solid nanoparticles, which undergo significant volume changes during K^+^ ion insertion/extraction. Owing to its layered structure and availability, graphite has been used as an anode material in metal-ion battery systems. However, during cycling it suffers from large volume variations, leading to low capacity and structural degradation. Several structural fabrication strategies have been developed, and engineered materials such as expanded graphite, polynanocrystalline graphite and activated carbon have been investigated to reduce these effects. However, securing long-term electrochemical and mechanical stability remains a major issue.^124,138,139^ In contrast, hollow carbon nanocages and porous frameworks better accommodate volume expansion and ion diffusion, resulting in prolonged cyclic stability and improved electrochemical performance. The graphitic carbon nanocages, when investigated at 35C and 0.1C, exhibit reversible capacities of 175 mA h g^−1^ and 221 mA h g^−1^, respectively.^140^

Using annealing and solvothermal methods, a novel composite material VSe_2_–CQDs was developed as an anode material. Coating VSe_2_ nano-sheets with CQDs in this approach helps to mitigate volume expansion and nano-sheet aggregation during charge/discharge. The overall capacity and K ion adsorption were enhanced because the material possesses abundant edge sites and oxygen-containing groups. After 200 cycles at 100 mA g^−1^, it delivers a high reversible capacity of 362 mA h g^−1^, while at 1000 mA g^−1^, a remarkable capacity of 225 mA h g^−1^ is achieved. Both achieved a coulombic efficiency of 99% (Fig. 8(a) and (c)). These impressive results make VSe_2_–CQDs one of the most promising anode materials reported so far for PIBs, with a straightforward design method.^141^ The capacity decreases from 362 mA h g^−1^ at 100 mA g^−1^ to 225 mA h g^−1^ at 1000 mA g^−1^, giving a retention of about 62%. This rate performance can be linked to the short diffusion pathway provided by the 0D CQD coating, rather than to any significant change in the conversion reaction of VSe_2_.

**Fig. 8:**
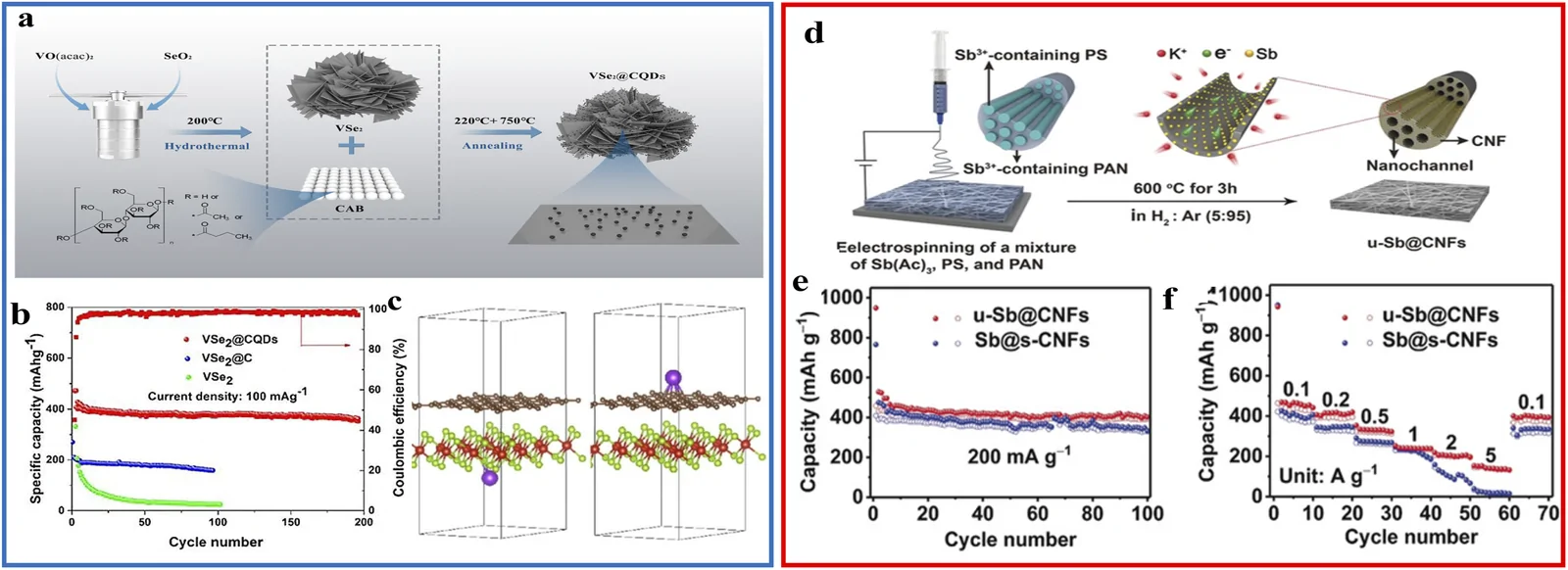
VSe_2_–CQD composite (a) synthesis (b) cyclic performance at 100 mA g^−1^ for PIBs (c) optimized DFT structure. This figure has been adapted from ref. 141 with permission from American Chemical Society, copyright 2022. u-Sb@CNFs (d) two-step synthesis of u-Sb@CNFs involving electrospinning of a PAN, Sb(Ac)_3_ and PS solution in DMF followed by calcination (e) cyclic performance at 200 mA g^−1^ (f) rate capability of Sb@s-CNFs and u-Sb@CNFs. This figure has been reproduced from ref. 144 with permission from Wiley-VCH, copyright 2019.

Alloy-based anode materials such as Ge, Sb, Bi, and P are widely known for their high theoretical capacity in PIBs. However, the major issues reported are volume expansion and mechanical stress.^142^ Reducing their dimensions to the 0D scale can therefore minimize both volume expansion and stress accumulation. It is necessary to design alloy-based anode materials at the nanoscale to improve their performance. To overcome such issues, a high-capacity Sb–CSN composite anode was developed. This material was designed by confining Sb within a conductive carbon sphere network for PIBs. First-principles calculations coupled with electrochemical measurements reveal multiple phase transitions from KSb_2_ to K_5_Sb_4_ during the intercalation/de-intercalation process.

The reported storage capacity was 551 mA h g^−1^ after 100 cycles at 100 mA g^−1^, fading by only 0.06% per cycle. It delivers a capacity of 504 over 220 cycles. For PIBs, it is currently considered the most promising anode material.^142,143^ Owing to their high theoretical capacity and optimal operating voltage, nanocomposites of antimony (Sb) are considered potential candidates for rechargeable batteries. To overcome the challenges faced by PIBs, a very straightforward approach is presented. The composites, referred to as Sb-CNFs (Sb nanocrystals embedded in carbon nanofibers), are employed not only as anodes but also to minimize the prominent limitations. A stable storage capacity of 225 mA h g^−1^ at a current density of 1 A g^−1^ is maintained over 2000 cycles (Fig. 8(d)–(f)). This unique design of Sb-CNFs leads to impressive rate performance, cyclic stability and superior electrochemical performance.^144^ Recently, pseudo-hexagonal Nb_2_O_5_ was reported for the first time as an anode material in PIBs. This material was fabricated using a one-step hydrothermal method, combining carbon nanotubes and Nb_2_O_5_ through glucose. It delivers a high capacity of 179 mA h g^−1^ at 0.3C and maintains 72 mA h g^−1^ at a high rate of 15C, demonstrating outstanding rate performance and cyclic stability.^145^ The good rate retention of Nb_2_O_5_ at 15C is notable because it is an intercalation-based material. This behaviour differs from the generally lower rate performance observed in conversion-type hosts for PIBs. The relatively small structural changes during intercalation may help K^+^ ions move more efficiently, even though K^+^ has a larger ionic radius.

Further studies have been carried out to improve the rate performance of PIBs. A pistachio-shuck MoSe/C nanostructure was fabricated to boost the performance of KIBs. To maintain structural stability during cycling, molybdenum selenide nanosheets are confined within a carbon shell. PMC offers dual advantages: it reduces hollow spaces, which improves packing density, and it also acts as a buffer for volume changes during K intercalation. PMC delivers an impressive capacity of 322 mA h g^−1^ at 0.2 A g^−1^ over 100 cycles and 226 mA h g^−1^ at 1 A g^−1^ over a long period of 1000 cycles in PIBs.^136^

Metal-based chalcogenides (MbCs) and metal-based oxides (MbOs) as electrode materials have real potential for PIBs, but they face several challenges, such as volume expansion and low conductivity. One effective technique to reduce such limitations is the design of 0D MbCs and MbOs, such as quantum dots, which mitigate volume changes and shorten ion diffusion pathways. A reported example is that Co_3_Se_4_ quantum dots enclosed by N-doped carbon have demonstrated superior performance compared with most reported materials. After 500 cycles, they delivered 100% capacity retention at 0.1 A g^−1^ and retained this performance at 0.5 A g^−1^ over 3200 cycles. They attained a stable capacity of 200 mA h g^−1^ even under high-rate conditions of 1 A g^−1^ after 10 000 cycles, as illustrated in Fig. 9(a) and (b). DFT calculations were performed to determine the theoretical capacity, and *in situ* X-ray diffraction and *ex situ* transmission electron microscopy confirmed its structural integrity and K intercalation/de-intercalation behaviour.^146^ Another reported material, SnS QDs supported on hollow mesoporous carbon fibres, achieved a stable capacity of 621 mA h g^−1^ at 1 A g^−1^ and 68.8% capacity retention over 1000 cycles.^137^ Similarly, FeS nanodots loaded on porous carbon exhibit a storage capacity of 232 mA h g^−1^ at 1 A g^−1^ after 3000 cycles. This study reveals that they exhibit fast ion transport, excellent stability and superior rate performance, making them potential anodes for PIBs.^135^ A composite of 0D TiO_2_ nanoparticles uniformly distributed on reduced graphene oxide was developed in 2019. TiO_2_ at the nanoscale enhanced K-ion transport owing to short diffusion pathways, whereas graphene oxide boosted the conductive network. This collaborative configuration demonstrates a high rate capacity of 107 mA h g^−1^ at a power density of 1000 mA h g^−1^. At the same current density, they achieved a capacity of 88.4 mA h g^−1^ after 1000 cycles. Despite their potential advantages, there is still room for improvement in terms of rate capacity and capacity retention under high loading conditions.^147^ Among these chalcogenide and oxide systems, the materials supported by a carbon shell or carbon fibers show better capacity retention during long-term cycling. In comparison, the TiO_2_/rGO composite has a lower overall capacity. These results suggest that the carbon matrix plays an important role in maintaining stability during cycling, rather than the 0D structure alone.

**Fig. 9:**
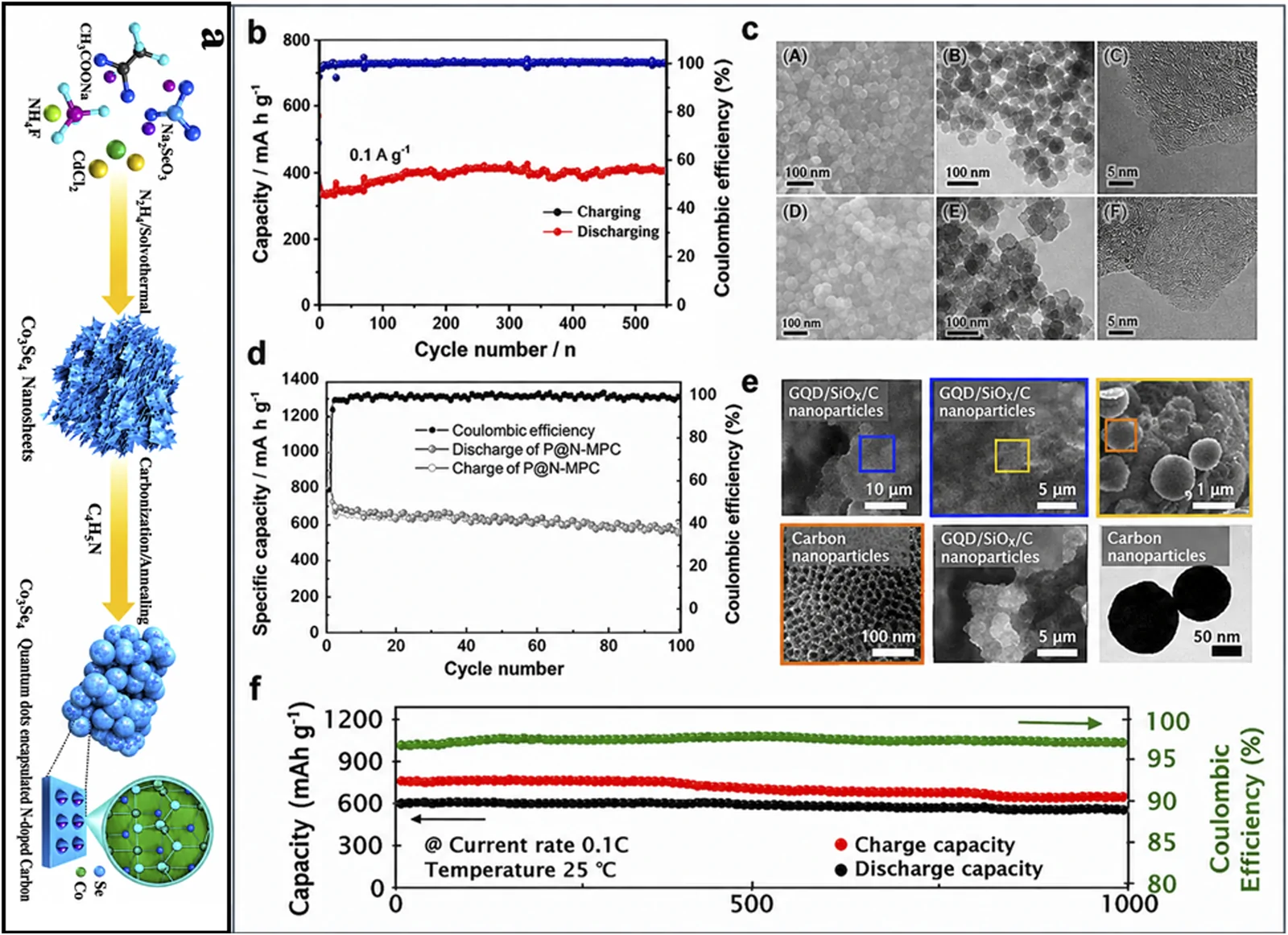
Co_3_Se_4_ quantum dots encapsulated by N-doped carbon (a) synthesis, (b) cycling at 0.1 A g^−1^ for PIBs. This figure has been reproduced from ref. 146 with permission from Wiley-VCH, copyright 2021. PN-MPC-0D composite (c) SEM/TEM/HRTEM N-MPC, (d) capacity and coulombic efficiency at 0.15 A g^−1^. This figure has been adapted from ref. 118 with permission from Wiley-VCH, copyright 2017. GQD/SiO_*x*_/C composite (e) TEM/SEM, (f) charge/discharge curves at 0.1C for LIBs. This figure has been reproduced from ref. 97 with open access permission from MDPI, copyright 2024.

For the first time, 0D CoS (conversion-type) nanoclusters in the form of QDs supported on graphene sheets were studied as a PIB anode material. The conductivity of graphene, combined with the high surface area of this composite, improved the structural integrity and energy storage ability of PIBs. This collaborative relationship between 0D CoS and the graphene matrix leads to a remarkable reversible capacity of 310 mA h g^−1^ over 100 cycles at a power density of 500 mA g^−1^. This study not only demonstrates the validity of CoS nanoclusters based on the carbon hybrid and quantum dot matrix but also contributes to a growing library of effective anode materials.^148^ Researchers developed a new anode material, referred to as SbSQ-MA (antimony quantum dots), with a supporting Ti_3_C_2_T_*x*_ MXene aerogel structure. This structure ensures buffering of volume changes, good dispersion and excellent electronic conductivity. High reversible capacities of 447 mA h g^−1^ and 246 mA h g^−1^ at 0.2 and 3.2 A g^−1^ deliver high-rate performance and cyclic stability. Even after 1000 cycles, it retains 94% of capacity. The results of XRD reveal that this material resists degradation over time, as the K–Sb phase is fully stable and reversible.^149^ Red P as an anode material for PIBs was first reported in the form of nanocomposites. It can be synthesized using 3D carbon nanosheets loaded with red P nanoparticles, resulting in red P–CN composites. This composite is reported to deliver 655 mA h g^−1^ reversible capacity while retaining 323 mA h g^−1^, along with a good rate capability of 2000 mA g^−1^. Volume changes are mitigated, and its structural stability surpasses that of other anodes for PIBs. By theoretical analysis (DFT), the theoretical capacity of a single redox reaction was calculated to be 843 mA h g^−1^.^150^ This value is a DFT-predicted capacity under idealized conditions and is therefore not directly comparable with the experimentally measured capacity of 655 mA h g^−1^ reported above, as discussed in Section 2.4.5. It is worth mentioning that potential improvements may be achieved by changing the dimensionality of the same material.

The design and potential of PIBs have been greatly improved by recent advances in 0D nanomaterials, which increase structural stability, conductivity and cyclic performance. Different classes of 0D materials, such as alloy QDs, carbon frameworks and nanocages, have shown promising results. Despite these promising results, some issues, such as poor long-term stability and capacity fading, need attention. Another unique class of materials, including clusters and molecular cages, is emerging as a potential anode candidate for next-generation batteries, although it is still new for PIBs.^152^ Continued efforts in interface engineering, design, mechanical understanding and synthesis techniques will help to bring them into real-world battery systems. Table 5 summarizes recent 0D anode materials for PIBs. Similarly to LIBs, the advantages of 0D materials for PIBs are reported in Table 4. A large number of anode materials for PIBs have been discussed, but such 0D anode materials may also face some limitations in practice. Bi@C, SnS QDs and antimony QDs do offer the best rate performance and cyclic stability, but a few limitations are possible. SEI formation can be triggered by the large surface area, which may reduce coulombic efficiency. Additionally, the synthesis of nanomaterials can be difficult and costly, which limits their commercial applications.^107^

**Table 5:** 0D anode materials for potassium-ion batteries (PIBs)

Anode material	0D structure	Capacity (mA h g^−1^)	Rate	Cycle life	Key advantage	Ref.
N/P co-doped CNPs	Carbon nanoparticles	270	0.2 A g^−1^	4000 cycles	DFT-guided design	123
Biomass-derived N/P	Carbon nanoparticles	193.3	100 mA g^−1^	—	Renewable source	151
Hollow carbon nano-cages	Nanocages	175/221	35C/0.1C	—	Better volume accommodation	140
Sb–CSN	Sb in carbon network	551	100 mA g^−1^	100 cycles	0.06% fade/cycle	144
VSe_2_–CQDs	QDs coated on nanosheets	362	100 mA g^−1^	200 cycles	>99% CE	141
Sb–CNFs	Nanocrystals in nanofibers	255	1 A g^−1^	2000+ cycles	Ultra-stable	144
Co_3_Se_4_ QDs	QDs in N-doped carbon	200	1 A g^−1^	10 000 cycles	100% retention at 500 cycles	146
FeS nanodots	Nanodots in porous carbon	232	1 A g^−1^	3000 cycles	Fast ion transport	135
MoSe/C (PMC)	Core-shell	322	0.2 A g^−1^	100 cycles	Improved rate	136
CoS nanoclusters	QD nanoclusters	310	500 mA g^−1^	100 cycles	Graphene support	148
Red P–CN	Nanoparticles	655	—	—	Good rate capability	150
SnP_3_/C	Nanocomposite	410	—	50 cycles	Improved stability	130
Sb QDs	Quantum dots	447	0.2 A g^−1^	1000 cycles	94% retention	149
TiO_2_ NPs/rGO	Nanoparticles	107	1000 mA g^−1^	1000 cycles	High rate	147
S-doped SnO_2_ QDs	QDs in carbon	102.8	10 A g^−1^	5000 cycles	Outstanding longevity	113
Bi@C	Nanoparticles	225	—	200 cycles	Suppressed expansion	130
SnS QDs	Quantum dots	621	—	1000 cycles	High capacity	137

### 3.4. Magnesium ion batteries (MIBs)

MIBs are gaining attention because of their high volumetric capacity of 3833 mA h cm^−3^, natural abundance and low cost.^153,154^ Despite these advantages, the limited availability of suitable electrode materials and sluggish ion transport restrict the progress of such batteries.^155,156^ To address such challenges, researchers have designed anode materials of different dimensionalities with extraordinary properties. Good cyclic stability along with a capacity of 350 mA h g^−1^ is exhibited by non-0D materials such as Bi nanotubes (Bi NTs). They retain around 303 mA h g^−1^ after 100 cycles. However, compared with their nanoparticle forms, their synthesis can be more complex and costly.^157^ Various 0D anode materials show better electrochemical characteristics, and research has consequently shifted towards 0D anodes. One notable anode material, SiOC/Sn nanobeads, was synthesized with C content for MIB anode applications. A combination of scanning electron microscopy, X-ray diffraction, elemental analysis, Raman spectroscopy and Fourier transform infrared spectroscopy (FTIR) was used for detailed characterization of the prepared ceramics. An initial discharge capacity of 198 mA h g^−1^ with retention of 144.5 mA h g^−1^ at 500 mA g^−1^ after 100 cycles, having a notable efficiency of 85.2% was revealed by performance results.^158^ The efficiency of 85.2% is important for MIBs because Mg^2+^ is a divalent ion and its insertion can face greater interfacial resistance than monovalent ions. The improved performance is likely related not only to the 0D Sn domains but also to the SiOC matrix, which helps reduce and accommodate surface reactions during cycling.

Manganese-based anodes are an excellent choice for MIBs because of their enhanced electrochemical performance and structural integrity at low cost.^121^ Bismuth-based anode materials are attracting researchers' attention because of their favourable magnesiation/demagnesiation behaviour. Researchers developed a new Sn/rGO anode material through a simple hydrothermal procedure followed by thermal processing. Anchoring Sn nanoparticles on reduced graphene oxide leads to better Sn particle distribution, reduced volume changes during charge/discharge cycles and improved electron and ion transport rates. At a current density of 15 mA g^−1^, the Sn/rGO anode material demonstrates a high reversible capacity of 362 mA h g^−1^ alongside excellent efficiency and rate performance. *Ex situ* and SEM analyses were performed to understand the behaviour of Mg demagnesiation.^122^ The comparison between the unsupported Bi nanotube and the Sn/rGO composite shows a similar trend to that observed in SIBs and PIBs. While the active material is mainly responsible for the capacity, the carbon support plays an important role in maintaining this capacity during repeated Mg^2+^ cycling.

The electronic, vibrational and structural features of B_24_N_24_ nanocapsules have been studied to investigate the effects of chalcogen and halogen encapsulation. The nanocapsules of B_24_N_24_ appeared to achieve an excellent cell voltage of about 3.46 V, while encapsulation with iodine raises it to 3.61 V, which is remarkable compared with the pristine value of 2.7 V.^171^ A recent first-principles study explores As_4_O_6_ inorganic molecular cages as potential anode materials for MIBs, predicting a comparatively high storage capacity of 1012 mA h g^−1^. The calculations indicate a low Mg energy barrier of about 0.13 eV and a diffusion coefficient of 1.13 × 10^−7^ m^2^ s^−1^. The material is predicted to be thermodynamically stable, with only 2% volume expansion, suggesting potential application in future energy storage systems. Similarly, the electronic, structural and storage properties of the cage-like molecular structure P_4_Se_3_ have been investigated by DFT for application in multivalent-ion batteries. The storage capacities of this material have been reported as 1484.84 mA h g^−1^, 519.69 mA h g^−1^, 1410.60 mA h g^−1^, 593.93 mA h g^−1^, and 74.24 mA h g^−1^ for sodium-, magnesium-, calcium-, aluminium- and zinc-ion batteries, respectively.^133^ These theoretical findings suggest that the materials could offer high electrochemical performance. However, to estimate the enhancement in electrochemical performance due to 0D molecular confinement, direct experimental validation of these materials under identical testing conditions is required, as discussed in Section 2.4.5. This distinction is particularly relevant in MIBs, where no molecular cage material has yet been investigated experimentally. The theoretical values above should be read as motivation for synthesis rather than as demonstrated performance.

Comparing 0D materials with their non-0D counterparts shows the benefits of reducing dimensionality for MIBs, and a few examples are highlighted here. Non-0D Sn-based anode materials experience volume changes and sluggish transport of Mg ions, resulting in capacity fading.^159^ On transition to 0D Sn nanoparticles (Sn@rGO), a high capacity has been achieved at 15 mA g^−1^.^122^ Excellent cycling efficiency and rate capability are also achieved. This improvement is attributed to low-dimensional properties, including low volume expansion and improved electrical conductivity. Furthermore, conventional non-0D SiOC/Sn nanocomposites show low Mg storage kinetics because of their limited surface activity. In contrast, 0D SiOC/Sn nanobeads achieve an initial discharge capacity of 198 mA h g^−1^ with a retained capacity of 144.5 mA h g^−1^ at 500 mA g^−1^ after 100 cycles, and a notable efficiency of 85.2% was reported. Through reversible alloying, conductivity and capacity are improved by increasing the Sn content, while Mg diffusion and storage are enhanced by the increased surface area in this study.^158^ Also, pure 0D anode materials such as P_4_Se_3_ and As_4_O_6_, discussed earlier, have no non-0D counterpart for comparison.

Benchmarking 0D against non-0D nanomaterials not only addresses the issues of bulk anodes but also highlights the advantages of 0D anode materials, namely high reversible capacity, good rate performance, excellent cycling stability and low volume expansion. Despite these advantages, 0D anode materials for MIBs face several challenges. The strong attraction between the host anode and Mg ions can hinder ion mobility and slow diffusion during cycling at a given current density. Electrolyte decomposition and SEI formation are promoted by the large surface area of 0D anodes, which reduces early-cycle efficiency.^122^ In practice, the synthesis of 0D nanostructures at the nanoscale is complex. Hybrid approaches may be promising for MIBs, but their experimental construction appears costly and difficult.^113,132^

### 3.5. Aluminium ion batteries (AIBs)

Aluminium-ion batteries have emerged as a strong competitor in modern energy storage owing to aluminium's abundant supply, high volumetric capacity, superior safety and cost-effectiveness.^160–162^ Unlike sodium-, lithium- and magnesium-ion systems, aluminium can deliver a higher energy density at low material cost, making it an attractive solution for large-scale energy infrastructure. However, the pathway to commercialization remains challenging owing to a shortage of suitable electrodes, slow ion migration and short cycle life.^163–166^ 0D nanostructures such as nanodots, nanospheres and hollow nanoparticles offer unique advantages, including expanded surface areas, shortened diffusion paths and adaptable surface properties. 0D anode materials work well in other monovalent and multivalent batteries, although research into 0D anode materials for AIBs is still at an early stage. The 0D materials discussed in this section store Al^3+^ through intercalation rather than metal deposition. Therefore, the aluminium metal anode itself does not act as the storage host.

To unlock their full electrochemical potential, researchers continue to undertake systematic investigations.^167^ Over the past five years, research on sustainable functional electrodes for AIBs has been carried out under thermal conditions. Among these, TiO_2_ has gained a reputation as a promising anode material for AIBs. One study reported that Al ions are reversibly intercalated into and deintercalated from TiO_2_ NPs. The introduction of graphene markedly boosts the electrochemical activity and diffusion coefficient within TiO_2_ by a factor of 44. The graphene–TiO_2_ composite exhibits an initial discharge capacity of 52 mA h g^−1^ at a high current density of 6.25 A g^−1^, far outperforming pristine TiO_2_. For the first time, a reversible TiO_2_ to Al_2_–TiO_2_ transformation triggered by Al^3+^ ion extraction and insertion was observed.^137^ Another titanium-based anode material was doped with nitrogen *via* a sol–gel process. Upon annealing, the N–TiO_2_ achieved 43.2 mA h g^−1^ at a current density of 3 A g^−1^. Kinetic evaluation showed rapid aluminium-ion diffusion, excellent electrical conductivity and low interfacial resistance. Nitrogen doping thus emerges as a promising modification strategy for TiO_2_ anodes in aqueous environments.^168^ Recent studies have highlighted TiO_2_ nanospheres (TiO_2_-NSs) as a viable anode material for AIBs. TiO_2_-NSs in this study were produced by hydrolysing a titanium glycolate precursor, followed by argon-atmosphere calcination and hydrothermal treatment. A large surface area of 179.9 m^2^ g^−1^ with a highly mesoporous morphology was achieved by these TiO_2_-NSs through the synthesis process.

TiO_2_-NSs morphological and structural analysis was carried out using FTIR spectroscopy, transmission electron microscopy and XRD. Owing to the large surface area, TiO_2_-NSs showed improved electrolyte efficiency, better electrolyte retention and superior rate capability, and facilitated faster electron/ion interaction. With an initial capacity of 183 mA h g^−1^ at 0.15C while maintaining 108 mA h g^−1^ even at 6.0C, TiO_2_-NSs demonstrated an excellent rate capability compared with TiO_2_–P25.^169^ The 44-fold increase in the diffusion coefficient after adding graphene shows that ion transport is not controlled by the 0D structure alone. The graphene matrix also plays an important role in improving ion transport, which agrees with the mechanism discussed in Section 3. Like other battery systems, the molecular P_4_Se_3_ architecture has also been investigated for AIBs using DFT-based first-principles calculations, which give a theoretical capacity of 593.93 mA h g^−1^ with a diffusion barrier of 0.33 eV.^133^ As discussed in Section 2.4.5, this DFT-predicted capacity has not yet been validated by experimental synthesis of a molecular-cage-based electrode. However, other lower-dimensional materials, especially 2D materials of different classes, have been experimentally tested in this regard. Most of the reported anode materials either exhibit long cycling with compromised rate capability or achieve excellent capacity but suffer from poor cycling stability.

Several reported aluminium anodes suffer from corrosion and hydrogen evolution, which limit the battery's performance. To resolve this issue, a recent study applied biomass-derived CQDs to aluminium anodes. The CQDs are so small that they act as an invisible, smart protective layer. They form a thin shield on the surface of the aluminium anode, blocking attack by the electrolyte on the metal surface and thereby preventing corrosion. This simple modification suppressed 91.3% of corrosion, demonstrating the promising potential of 0D nanomaterials for AIB anodes. It should be noted that in this application the CQDs function as a modifier on the aluminium metal anode and are not used as a storage host. Because of this, their role cannot be directly compared with the capacity values mentioned earlier for TiO_2_-based intercalation hosts. For this reason, they are listed as metal deposition interface modifiers in Table 6. Unlike LIBs, SIBs and PIBs, only a few pure 0D anode materials have been reported for AIBs, indicating a clear research gap in this field. Several 0D nanomaterials, however, have been used together with aluminium anode materials, enhancing the electrochemical properties of AIBs.

**Table 6:** Benchmarking of 0D anode materials across battery systems

Material	0D structure/battery type	Class	Capacity (mA h g^−1^)	Cycle life	Rate performance	Ref.
π-Conjugated molecule-bridged Si-QD clusters	Quantum dots/LIB	Alloying	1957 (initial)	100 cycles	200 mA g^−1^	100
SiO_2_ QDs/rGO nanocomposite	Quantum dots/LIB	Alloying	865 (initial)	60 cycles	200 mA g^−1^	99
GQD/SiO_*x*_/C composite	Graphene quantum dots/LIB	Alloying	595 (after 100 cycles)	100 cycles	Moderate	97
SnO_2_ QDs/N-doped C	Quantum dots/LIB	Conversion	1255 (after 100 cycles)	100 cycles	Not specified	96
Graphene-encapsulated Co_3_O_4_ QDs	Quantum dots/LIB	Conversion	1785 (initial)	100 cycles	1000 mA g^−1^	103
Coal-based GQDs/α-Fe_2_O_3_ nanocomposite	Graphene quantum dots/LIB	Conversion	1582.5 (initial)	110 cycles	1 A g^−1^	102
Si nanoparticles/SiNG composites	Nanoparticles/LIB	Alloying	1568.9 (SiNP, initial); 1780.7 (SiNG, initial)	100 cycles	8400 mA g^−1^	93
Co_3_Se_4_ QDs	Quantum dots/PIB	Conversion	200 (at 1000 mA g^−1^)	10 000 cycles	Moderate	146
SnS QDs in hollow carbon nanofibers	Quantum dots/PIB	Conversion	621 (at 1 A g^−1^)	1000 cycles	1 A g^−1^	148
MoSe_2_/C core–shell (PMC)	Core-shell 0D/PIB	Conversion	322 (at 0.2 A g^−1^)	100 cycles	0.2 A g^−1^	147
CoS QD nanoclusters on graphene	Quantum dot nanoclusters/PIB	Conversion	310 (at 500 mA g^−1^)	100 cycles	500 mA g^−1^	149
Red P–CN composites	Nanoparticles/PIB	Alloying	655 (at 50 mA g^−1^)	—	—	161
TiO_2_ NPs/rGO (MXene-derived)	Nanoparticles/PIB	Intercalation	107 (at 100 mA g^−1^)	1000 cycles	High	158
3D–0D graphene–Fe_3_O_4_ QD hybrids	Quantum dots/SIB	Conversion	525 (at 30 mA g^−1^); 312 (after 200 cycles)	200 cycles	10 mA g^−1^	115
Carbon quantum dots (CQDs) from glucose	Quantum dots/SIB	Alloying	323.9 (at 0.5C)	500 cycles (72.4% retention)	0.5C	120
N/S co-doped sheet-like graphitic CQDs	Quantum dots/SIB	Alloying	155 (at 5C)	4000 cycles	5C	121
S-doped SnO_2_ QDs in N,S-codoped carbon fiber network	Quantum dots/SIB	Conversion	141 (at 20 A g^−1^); 237 (at 5 A g^−1^ after 10 000 cycles)	10 000 cycles	20 A g^−1^	113
VN QDs anchored on carbon nanofibers	Quantum dots/SIB	Conversion	230.3	500 cycles	0.5 A g^−1^	124
Sn_2_S_3_ QDs in N,S-codoped carbon fiber network	Quantum dots/SIB	Conversion	300 (at 10 A g^−1^); 250 (at 20 A g^−1^)	1000 cycles	10–20 A g^−1^	132
SiOC/Sn nanobeads	Nanobeads (0D)/MIB	Alloying	198 (at 50 mA g^−1^)	100 cycles	500 mA g^−1^	169
Sn/rGO	Nanoparticles/MIB	Alloying	362 (at 15 mA g^−1^)	—	15 mA g^−1^	122
B_24_N_24_ nanocapsules	Nanocapsules (0D)/MIB	Intercalation (theoretical)	536 (theoretical)	—	—	189
P_4_Se_3_ molecular cage	Molecular cage (0D)/MIB	Intercalation (theoretical)	519.69 (theoretical)	—	—	187
P_4_Se_3_ molecular cage	Molecular cage (0D)/AIB	Intercalation (theoretical)	593.93 (theoretical)	—	—	187
TiO_2_ nanospheres	Nanospheres (0D)/AIB	Intercalation	183 (at 0.15C)	—	0.15C	181
N–TiO_2_	Nanoparticles/AIB	Intercalation	43.2 (at 3 A g^−1^)	—	3 A g^−1^	180

Unmodified and powdered aluminium foil anodes face intrinsic electrochemical limitations that make Al plating difficult. Aluminium readily forms a dense, thin passivation layer on its surface in both aqueous and non-aqueous environments, which behaves as an insulating layer. When bulk aluminium is used as an anode material, this layer, combined with corrosion and hydrogen side reactions, limits the cycle life and practical capacity.^170^ To resolve these issues, researchers have begun modifying aluminium by coating it with nanomaterials and bismuth-like coatings to remove the insulating barrier and promote smooth Al-ion diffusion. As a result, the surface area increases and the kinetic barrier decreases, which is more effective than the traditional approach.^171^ The anode materials reported for AIBs show major advantages, but they also have material-specific drawbacks. For example, B_24_N_24_, TiO_2_ nanocomposites and P_4_Se_3_ achieve high capacity and large surface area, but the larger size of Al ions may cause slow diffusion and desolvation when passing through electrodes. A high surface area may result in side reactions and electrolyte decomposition.^107,169^ Furthermore, the large-scale manufacturing of cage-like materials is difficult.

The AIB evidence follows the MIB pattern discussed in Section 3.4. Data on capacity and rate capability are mostly available for the TiO_2_ family of intercalation hosts. There is one example of a non-storage interfacial application, namely CQD corrosion protection, and one result from DFT studies of molecular cages. Unlike for MIBs, the TiO_2_ results include a controlled comparison: graphene TiO_2_ compared with TiO_2_ under the same conditions. This is why the claim for the AIB intercalation class in Section 4.1 is better supported than the MIB one, although research on both chemistries remains limited overall.

### 3.6. Zinc ion batteries (ZIBs)

Zinc-ion batteries offer high theoretical capacity, low production cost and an eco-friendly nature, and ZIBs have emerged as strong contenders for large-scale energy storage. Although divalent Zn ions boost energy density, their high charge density imposes severe constraints on ion diffusion kinetics, encouraging dendrite formation and side reactions and causing sluggish electrochemical behaviour.^172–178^ These inherent challenges call for the development of host materials with high mechanical strength and low-barrier pathways for Zn transport.^178–180^ Higher-dimensional materials such as 1D, 2D and 3D materials frequently suffer from weak structural stability, anisotropic ion transport and sluggish interlayer movement. In contrast, 0D nanostructures and their integration into previously reported anode materials offer large surface exposure, superior structural resilience, isotropic pathways and exceptional durability, making them well suited to meeting anode challenges. One of the most prominent challenges faced by higher-dimensional anode materials is dendrite formation, which can be effectively alleviated through the use of 0D anodes for ZIBs.^181^ It is important to note that in ZIBs, zinc metal itself acts as the anode. The 0D materials discussed in this section (CQDs, hydrophilic QDs, N/S-CQDs and MXene QDs) do not store Zn^2+^ through intercalation, alloying or conversion reactions. Instead, they serve as additives or interface coatings that control Zn nucleation and suppress dendrite growth. Their role is not capacity-based, so their performance should not be directly compared with the ion storage capacities of LIB, SIB or PIB anodes, as noted in Section 3.

By contrast, P_4_Se_3_ and ZnO QDs/COF are proposed as storage hosts and are classified accordingly. Owing to their affordability and strong safety features, aqueous ZIBs offer significant potential for large-scale stationary energy storage. The uneven plating of Zn ions leads to dendrite formation, limiting the practical use of AZIBs. In this study, nanocrystalline and hydrophilic carbon QDs were synthesized and incorporated into the electrolyte to address the dendrite problem. The high graphitization of the CQDs increases conductivity and reduces the polarization voltage and interface impedance, while abundant hydrophilic groups facilitate uniform Zn deposition, thereby enhancing the rate capability. The hydrophilic nature, coupled with the nanocrystalline structure of the CQDs, lowers Zn^2+^ nucleation barriers and improves Zn stripping/plating kinetics, allowing Zn//Zn cells to operate stably at 1 mA cm^−2^ for over 1000 h and showing low voltage gaps of 210.2, 156.4, 120.5 and 98.3 mV across 1–4 mA cm^−2^. CQD-modified Zn//MnO_2_ cells retain 74% of their capacity after more than 500 cycles, and Zn//Cu cells exhibit an average coulombic efficiency of 99.7% at 0.5 and 1 mA cm^−2^. This 0D material focuses on electrochemical and morphological behaviour, and theoretical capacity is not reported.^182^ No work has yet been reported on Zn^2+^ storage in these 0D materials, because their role is not capacity-based. In studies of these materials, the key performance measures are cycling time and efficiency rather than storage capacity. This matches the classification in Section 3, where 0D materials in ZIBs are grouped under the metal deposition interface modifier class, not the ion storage class. Hydrophilic QDs are another approach introduced to modify the nucleation process, promoting uniform deposition and preventing dendrite formation. Without QDs, AZIBs suffer from zinc dendrite growth and poor cycling stability, which hinder their practical use. This enhanced anode material lowered the polarization voltage from 80 to 50 mV (at 0.8 mA cm^−2^), prolonged cycling stability to 2200 h and maintained a capacity of 164.3 mA h g^−1^ after 600 cycles with a vanadium cathode.^183,184^ Another reported material, nitrogen and sulphur co-doped CQDs, serves as a zincophilic additive to promote uniform zinc deposition. Rich in electronegative groups, NS-CQDs attract Zn^2+^ ions and deposit them on the electrode surface, promoting parallel growth of the (002) crystal plane. Zn deposition along the (002) direction prevents dendrite formation. The long-lasting plating/stripping behaviour allows the anode material to work well at a balanced N/P ratio of 1.05, with a good energy density of 144.98 Wh kg^−1^.^185^ MXene QDs self-heal by forming a Zn layer on their surface. They delivered cycling stability for 3700 h with 99% efficiency and corrosion protection for 4000 h. When combined with cathodes such as I_2_ and MnO_2_, they exhibit excellent full-cell cycling.^186^ P_4_Se_3_ molecular cages offer a stable capacity of 74.24 mA h g^−1^ and a 0.71 eV diffusion barrier.^187^ They are also considered suitable anode materials for multivalent batteries. In contrast to the interfacial modifiers discussed above, P_4_Se_3_ is the only entry in this section proposed as a true Zn^2+^ storage host and is classified accordingly in Table 6. However, the same DFT limitation applies here as in Sections 3.2, 3.4, and 3.5. The reported value of 74.24 mA h g^−1^ is based on DFT calculations and has not been experimentally validated. Therefore, it should not be directly compared with the experimentally measured capacities reported for the LIB, SIB, and PIB systems.

Alkaline zinc-based batteries have attracted considerable attention because of their high safety, high energy density and availability. ZnO QDs combined with covalent organic frameworks (COFs) work well in Zn-based battery systems. With the Ni (OH) _2_ cathode in a Zn/Ni battery, it delivers a reversible capacity of 540 mA h g^−1^. It maintains a capacity retention of 87% over 4000 cycles and 90% over 2000 cycles at 12C. Considering the advantages of 0D anode materials for ZIBs, their limitations are discussed separately here. 0D anode materials for ZIBs, such as FeS and Co_3_O_4_ QDs, face cracking, stress build-up and dissolution of active material due to the fast insertion of Zn ions.^184^ Side reactions with the electrolyte may be triggered by the large surface area, leading to shorter cycle life and lower coulombic efficiency. Reproducibility and large-scale manufacturing are also common issues. True 0D nanomaterials and 0D-modified systems are two approaches that complement each other in enhancing battery performance.

Overall, the data on ZIBs support the argument made in Section 4.1. With the exception of the ZnO QD–COF composite, the benefits of the 0D materials in this section do not come from higher storage capacity. Their advantages are kinetic: reduced dendrite growth, lower nucleation overpotential, and better cycling stability during plating and stripping. Placing these materials in the same 0D anode category as capacity-driven LIB, SIB, and PIB anodes would therefore mix two different types of performance. To avoid this confusion, we discuss the 0D materials used in zinc-based systems separately, in Section 3.6 and Table 6.

## 4. 0D anode materials benchmarking across battery systems

The benchmarking data in Table 6 reveal three general trends. First, 0D anode design is governed by a capacity–stability trade-off. Si-based QDs in LIBs achieve the highest capacities (1957 mA h g^−1^) but suffer rapid decay, whereas Co_3_Se_4_ in PIBs offers a lower capacity (200 mA h g^−1^) over a very long lifetime (10 000 cycles).^99,100^ This dichotomy shows that the 0D architecture alone is not sufficient; the balance between energy density and lifespan is determined by matrix integration. Second, high performance cannot be achieved without conductive matrices. Graphene, carbon structures and carbon fibre mesocrystals are found in the best 0D anodes.^99,103,148,188^ These supports serve a dual purpose: electronic percolation to overcome low intrinsic conductivity, and mechanical buffering of volume expansion, which is essential for both alloying (Si, Sn) and conversion (oxides, sulfides) materials. Third, the choice of material depends on the charge carrier. LIBs exploit alloying materials in a 0D morphology, which inhibits pulverization. PIBs make use of selenides and sulfides (Co_3_Se_4_, SnS, CoS) whose frameworks are stable enough to accommodate the larger K^+^ ion. SIBs rely on carbon-based QDs incorporated in a carbon fibre network, balancing conductivity and structural flexibility for Na^+^ intercalation.^113,117,188^ Multivalent systems (Mg^2+^, Al^3+^, Zn^2+^) are still under-explored experimentally, and most candidates have so far been analysed only by DFT.^133,187^ A new paradigm in ZIBs is that 0D materials (CQDs, MXene QDs) now act as interface modifiers rather than active materials, allowing dendrite suppression and self-healing of interphases.^175,179^ Inconsistency in reporting cycle life (cycle number, retention percentage or hours of operation) hinders strict cross-comparison.

The DFT-predicted values are upper bounds under idealized single-particle, defect-free conditions (see Section 2.4.5) and are not directly comparable to experimentally measured capacities in the same column, which reflect composite-electrode, multi-cycle performance under realistic SEI and agglomeration losses.

A major challenge in benchmarking 0D anodes is the inconsistency in reporting metrics. Some studies report initial capacity, others report retained capacity after hundreds or thousands of cycles, and others report theoretical values from DFT. Cycle life is reported variously as cycle number, retention percentage, or operating hours. This heterogeneity limits strict cross-comparison and underscores the need for standardized reporting protocols in the field.

### 4.1 Scope of the 0D advantage across chemistries

The data given in Table 6 do not support a universal performance advantage for 0D architectures across all battery chemistries. They suggest instead that the size and source of the benefit of 0D confinement vary considerably with the ion chemistry, the storage mechanism, and the electrode configuration. A key question is whether the reported improvements come from 0D confinement itself or from the carbon matrix, doping, or electrolyte optimization that often accompany 0D materials. Few studies isolate the effect of dimensionality alone, and this is a significant gap in the literature.

In monovalent systems, several 0D architectures show high capacity, rate capability, or cycling stability, consistent with the proposed benefits of shorter diffusion pathways and better accommodation of structural changes. Bridge-structured Si QDs in LIBs, for example, deliver an initial capacity of 1957 mA h g^−1^, although only about 63% of it is retained after 100 cycles.^110^ S-doped SnO_2_ QDs in SIBs deliver 141 mA h g^−1^ at 20 A g^−1^ and 237 mA h g^−1^ at 5 A g^−1^ after 10 000 cycles.^123^ Co_3_Se_4_ QDs in PIBs retain 200 mA h g^−1^ at 1 A g^−1^ after 10 000 cycles.^158^ In multivalent systems, the available results combine theoretical predictions with a few experimental demonstrations, so firm conclusions must wait for more experimental work. DFT predicts high capacities of 536 mA h g^−1^ for B_24_N_24_,^171^ 519.69 mA h g^−1^ for P_4_Se_3_ in MIBs, and 593.93 mA h g^−1^ for P_4_Se_3_ in AIBs,^133^ but these are theoretical upper bounds (Section 2.4.5) and not directly comparable with measured values. The early experimental results, such as 362 mA h g^−1^ for Sn/rGO in MIBs^122^ and 183 mA h g^−1^ for TiO_2_ nanospheres in AIBs,^182^ are encouraging, but systematic comparisons under identical conditions have yet to be made.

The role of 0D materials in Zn systems is different. In quantum-dot architectures such as CQDs and MXene QDs, the main contribution is at the interface, not ion storage. CQDs allow Zn//Zn cells to run stably for over 1000 h at 1 mA cm^−2^, and MXene QDs provide 3700 h of cycling stability with 99% efficiency. Taken together, the evidence points to a chemistry-specific, mechanism-dependent advantage for 0D architectures, not a universal one across all battery systems.

## 5. Future directions

Combined advances in scalable synthesis, interface engineering, multivalent systems, mechanistic understanding and practical metrics are required to translate 0D anode materials into next-generation batteries. The key limitation is scalable synthesis, since existing quantum dot syntheses rely on low-yield batch processes; continuous-flow reactors, microwave-based synthesis and spray pyrolysis can provide routes to high-throughput synthesis and size control.^64,67^ Interface engineering is also very important because the high surface area of 0D materials promotes parasitic reactions that result in unstable solid–electrolyte interphase formation; strategies to counter this behaviour are promising (conformal layers deposited by atomic layer deposition, *e.g.*, Al_2_O_3_ and TiO_2_, and *operando* characterization to understand SEI evolution).^100^ Beyond lithium-ion systems, multivalent systems (Mg^2+^, Al^3+^, Zn^2+^) are promising, since 0D structures can provide a unique way to address slow diffusion kinetics and large volume changes; systematic experimental assessment of molecular cages and quantum dots for multivalent-ion storage is a critical area.^40,190–192^ The new paradigm of 0D materials as multifunctional additives rather than primary active materials is also worth developing, and dual-function additives that improve transport, reduce dendrites and contribute to storage have the potential to transform systems in which interface stability is becoming a bottleneck.^182,186^ Mechanistic understanding should be enhanced by *in situ* transmission electron microscopy to visualize lithiation dynamics in single particles, *operando* X-ray methods to determine structural evolution, and multiscale modelling that bridges atomic-scale DFT and continuum simulations to enable predictive design. Lastly, bridging the gap between academia and industry should involve the use of practical metrics such as areal capacity (mA h cm^−2^), full-electrode energy density, low-temperature operation and calendar life, rather than reporting only the specific capacity of the active material. Solving these interdependent issues will establish 0D anode materials as a viable route towards next-generation, high-capacity, long-life battery technology.

## 6. Conclusion

0D nanostructures have emerged as promising anode materials for advanced monovalent and multivalent ion batteries. Their distinctive properties, such as quantum confinement effects, high surface-to-volume ratio and the ability to accommodate volume changes, allow 0D nanomaterials to facilitate rapid ion transport, better electrochemical kinetics and more stable cycling than other non-0D materials. In this review, the reported 0D anode materials for diverse battery systems were explored, and their potential, existing limitations and current solutions were highlighted. Ongoing research has already addressed several obstacles through the introduction of surface coatings, composite integration, low-cost synthesis and engineered hybrids. Continued research is needed to improve synthesis routes, enhance cycle life, reduce production costs and advance electrode engineering. Overall, true 0D nanomaterials and 0D-modified systems are not just effective alternatives to non-0D and conventional counterparts but also key enablers of stable, reliable and long-lasting high-performance energy storage devices.

## Conflicts of interest

The authors declare that they have no known competing financial interests or personal relationships that could have appeared to influence the work reported in this paper.

## Data Availability

There is no additional data associated with this article.
